# Influences of Depulping, Pod Storage and Fermentation Time on Fermentation Dynamics and Quality of Ghanaian Cocoa

**DOI:** 10.3390/foods13162590

**Published:** 2024-08-19

**Authors:** Stefanie Streule, Amandine André, Susette Freimüller Leischtfeld, Karin Chatelain, Elodie Gillich, Irene Chetschik, Susanne Miescher Schwenninger

**Affiliations:** ZHAW Zurich University of Applied Sciences, Institute of Food and Beverage Innovation, 8820 Wädenswil, Switzerlandamandine.andre@zhaw.ch (A.A.); susette.freimueller@zhaw.ch (S.F.L.); karin.chatelain@zhaw.ch (K.C.); elodie.gillich@zhaw.ch (E.G.); irene.chetschik@zhaw.ch (I.C.)

**Keywords:** cocoa beans, cocoa liquor, key aroma compounds, key taste compounds, post-harvesting, pulp-preconditioning, quality assessment, sensory analysis

## Abstract

This study investigated the impact of the depulping of cocoa beans after pod opening, as well as the influences of pod storage (PS) and fermentation time on the fermentation dynamics and the overall quality of beans and liquors made thereof. Twelve variations were conducted in three experimental runs (with/without depulping; 1-/3-day PS; and fermentation times of 3, 4, 5, 6 or 7 days). Fermentation dynamics (e.g., temperature and pH) and the quality of dried beans (e.g., cut-test and fermentation index) and liquors (sensory assessment, quantification of cocoa key-odorants and tastants) were investigated. It was demonstrated that 17–20% of cocoa pulp, relative to the total bean-pulp-mass weight, could be mechanically removed without negatively affecting the bean quality. No significant differences were found in the percentages of well-fermented beans after 5–6 days fermentation with 1-day PS, resulting in 49 ± 9% with, and 48 ± 12% without depulping. There were no significant differences in key tastants present in the liquors; however, significantly less volatile acids and esters were found when liquors were produced from 5–6 day-fermented depulped beans, with 1-day PS, without negatively affecting the sensory profiles. This strategy allows producers to maximize the cacao fruit’s value by integrating part of the pulp into the cocoa value chain.

## 1. Introduction

Ghana is considered the second largest cocoa producer, after Côte d’Ivoire, with an estimated market share of 14% of global production in the 2021/22 crop year [1]. Over 800,000 smallholder farm families are involved in cocoa farming [2] across seven regions in Ghana, including Brong Ahafo, Western North, Western South, Eastern, Ashanti, Central and Volta [3]. The main crop season is from September to January, while the light crop season lasts from February to August [4].

After harvesting, cacao pods are stored for 1 to 7 days, and sometimes even longer, before pod-breaking [5]. However, the Cocoa Research Institute of Ghana recommends that pod breaking should be performed within 2–3 days [6]. This practice is used by 87.8% of Ghanaian farmers, which could be the reason for the typical flavor profile of Ghanaian cocoa [5]. It also shows the potential of accelerating the fermentation (e.g., by quicker increase in temperature) and improving the end quality of beans (e.g., more well-fermented beans in cut-test, and reduced acidity in the cocoa beans), as previously investigated in Ghana [7,8], Ecuador [9], and Malaysia [10,11,12]. After pod storage and breaking, the fermentation takes place, commonly in the form of heaps of cocoa beans; this is a customary practice in West Africa, and is suitable for smallholder farmers [13]. The heaps are formed by placing cocoa beans (300–2000 kg) on fresh plantain leaves and covering them with further leaves. Heap fermentation in Ghana lasts, optimally, for 6 days, with turning steps in 48 h intervals, a practice followed consistently by almost all farmers [14,15]. Nevertheless, various studies have investigated the potential of reducing the fermentation time (e.g., with combinations of alternative fermentation methods) [16,17,18] and the influence of turning on fermentation dynamics and end quality (e.g., [18,19,20]). Drying of beans is then effected on raised mats, with frequent turning, for about 7–14 days, until reaching a moisture content of 7.5% [14,15].

Currently, approximately 80% of the cacao fruit remains unused; this portion comprises the cocoa husk, cocoa bean shells and cocoa pulp, which could be utilized for value-added purposes in applications in various food industries. Therefore, the significance of cocoa by-products is increasing, driven by social and environmental concerns surrounding their disposal. For instance, disposal of cocoa husks on the farms without proper treatment can lead to plant diseases. By adding value to the whole cacao fruit, opportunities for additional income for farmers can be generated [21,22,23]. Various studies have investigated the potential uses of by-products, one example being a use to improve the cocoa pulp’s shelf-life [23], or the use of extraction technologies to derive bioactive compounds from cocoa processing residues in Ghana in order to reduce waste and increase the utilization of cocoa processing [24].

The cocoa pulp surrounding the raw cocoa beans has been shown to play an important role during fermentation [25,26,27]. The bioconversion of the pulp, first by yeast, and then by lactic and acetic acid bacteria, leads to the formation of the respective organic acids. These acids permeate the cocoa seed tissue and help to create the right environment for the enzymatic release of aroma and flavor precursors such as free amino acids and reducing sugars, while also triggering the biochemical transformation of the flavanols that leads to reductions in bitterness and astringency [25,28]. Moreover, cocoa pulp has been proven to influence the chocolate aroma profile through the absorption of the aromatic compounds by the cocoa cotyledon during the fermentation process [28,29]. Although cocoa pulp is important in the processing of cocoa beans, studies have shown that a mechanical removal of 10–30%, determined by weight based upon the weight of the fresh beans, is possible without negatively affecting the quality of the fermented and dried beans [25,30,31]. This partial removal of pulp can even be advantageous in improving the flavor quality of the beans, while an excess of pulp can lead to overly acidic beans [25,32]. Furthermore, it also can improve the drying time [33]. Cocoa pulp is therefore an interesting by-product, containing 10–13% sugars (sucrose, glucose and fructose), pentosans and pectins (1–4%) and citric acid (1–3%) [34]. Often consumed as fresh juice, cocoa pulp has more recently been used by the chocolate industry to produce “fruit-to-bar” chocolates, in which the added sugar is replaced by cocoa pulp [35]. For this reason, part of the pulp could be mechanically removed before fermentation and used for other purposes, resulting in an added value for the cocoa value chain. However, the impact of depulping on selected, well-known cocoa and chocolate key odorants and tastants identified in cocoa liquors [36,37,38] has, to our knowledge, never been studied.

With rising competition and production costs in Ghana, finding innovative and cost-effective methods to enhance cocoa quality [16] and also to valorize the whole cacao fruit is crucial. An effective approach involves combining (1) depulping to add value, (2) testing the potential reduction of fermentation time to save time and costs, and (3) examining pod storage, a common practice in Ghana, to enhance overall quality and maximize the fruit’s value.

Therefore, the objective of this comprehensive study was to combine these three parameters, evaluating the effect of the depulping on fermentation dynamics and end quality of cocoa beans and liquors made thereof. An important aspect was to determine whether a portion of the pulp can be removed before fermentation and utilized for other purposes, thereby enhancing the value of the cacao fruit without negatively affecting the quality of the depulped, fermented and dried beans and their resulting liquors.

This was investigated by conducting three experimental runs with a total of 12 variations (differing in pod storage time, in depulping and in fermentation times). Our research included monitoring of the fermentation process, focusing on key aspects such as the pH of the cotyledon and the temperature, sugars and organic acids, as well as an assessment of the viability of the cocoa embryo. Additionally, analyses of dried beans, including sugars and organic acids; cut-tests; and the determinations of the fermentation index and the pH of the dried cotyledon were conducted. The quantification of selected key tastants (flavanols DP 1–4 and alkaloids) and selected key cocoa aroma compounds, together with sensory analysis of the cocoa liquors, allowed the investigators to evaluate the impacts of depulping on the molecular flavor composition and sensory properties of the liquors, as well as to understand potential influences of fermentation variations on the final product quality.

## 2. Materials and Methods

### 2.1. Overview of Experiments

The study was carried out over three experimental runs (A, B and C) conducted in the Central Region of Ghana, and spanning from August to September 2022 (A), September to October 2022 (B) and from September to November 2023 (C). In experimental runs A and B, fermentations were performed in two independent runs each, with two dependent repetitions per run. For run C, three independent runs were conducted, each with two dependent repetitions. Variations across the experimental runs included the investigation of pod storage durations (1 or 3 days), depulping processes (with or without), and fermentation times (3, 4, 5, 6 or 7 days) (Table 1).

### 2.2. Pod Storage, Depulping, Fermentation, Drying and Sampling

**Figure 1:**
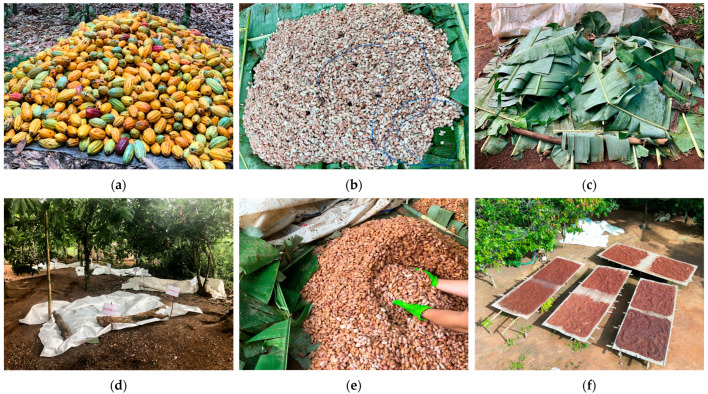

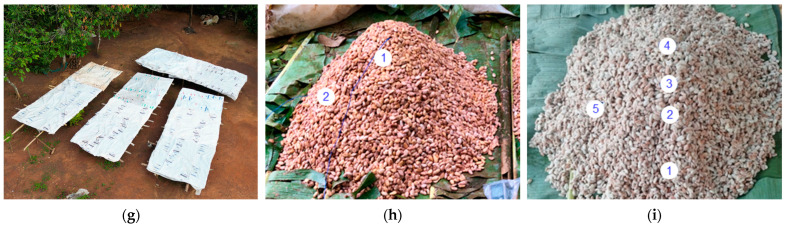
(**a**) Heaps during pod storage; (**b**) heap on d0 of fermentation, with holes for aeration and drainage beneath plantain leaves; (**c**) heaps covered with plantain leaves; (**d**) heaps covered with plastic sheets; (**e**) manual turning of the beans; (**f**) beans drying on bamboo mats; (**g**) beans covered with plastic during rainfall and during the night; (**h**) points 1 and 2 indicate sampling positions (1: in center of the heap, 2: at the side, within the middle of the heap) during experimental runs A and B; (**i**) points 1–5 indicate sampling positions (1, 2, 4 and 5: in the center, within the middle; 3: from the surface) during experimental run C.

Cacao pods were harvested and piled upon plantain leaves in order to protect the pods from dirt (Figure 1a). Following the experimental design, the pods were stored for two different durations: a 3-day storage period before opening, or a 1-day storage period with opening the next morning. On the day designated for opening the pods (=d0), cocoa beans were manually extracted from the pods, ensuring the removal of the placenta. These extracted cocoa beans were then collected in sealed plastic buckets. For variations with depulping (D1), the beans were processed by Koa Impact Ghana Ltd. (Akrofuom, Ghana) in a destoning machine. Between 17 and 20% of cocoa pulp based on the total bean-pulp-mass weight was removed.

Then, eight heaps of 90–100 kg of cocoa bean-pulp mass were formed on plantain leaves (heap sizes of approx. 115 × 118 × 35 cm). Holes were formed in the mass for aeration and drainage of the pulp (Figure 1b); the heaps were then covered with plantain leaves and weighed-down with wood (Figure 1c), and plastic sheets were stretched over the heaps (Figure 1d).

During the fermentation process, the heaps were turned 1–2 times, using gloves (Figure 1e), in d2 and d4, except for run 1 from experimental run A, in which the beans were only turned on d3. After the fermentation process, the drying process was started by spreading the beans on bamboo mats (Figure 1f) and turning them by hand several times (on average, every 1–2 h) a day. In case of rain and at night, beans were covered with the mat and plastic sheets (Figure 1g). The drying lasted until the moisture content was below 7% (measured using a mini GAC moisture tester, Dickey-John, Auburn, IL, USA), which corresponded to a duration of 10–14 days of drying.

Sampling was performed daily using disinfected gloves. Samples were taken from different positions of the heap during fermentation (Figure 1h,i) and mixed before the analyses were carried out (Section 2.3). Samples of dried beans were stored at −20 °C for HPLC analyses of sugars and organic acids (sucrose, glucose, fructose, citric acid, lactic acid and acetic acid). Beans used for the fermentation index assessment and cocoa liquor production were stored at room temperature before being transported to Switzerland.

### 2.3. Analyses during Fermentation and Drying, and of the Dried Cocoa Beans

#### 2.3.1. Measurements of Pulp Content and pH of Cotyledon

The pulp content at the start of fermentation was determined as described by Romanens et al. [39], by weighing 20 cocoa beans before and after depulping, and using tissue paper for the depulping. Measurements were performed one to three times for each heap. Cotyledon pH was measured daily during fermentation and at the end of drying according to the description of Romanens et al. [40], using a portable pH-meter (VWR International, Radnor, PA, USA). In experimental runs A and B, pH values of each heap were measured; in run C, the pH was determined from a mixed sample of both heaps.

#### 2.3.2. Measurement of Temperatures in Fermentation Heaps

**Figure 2:**
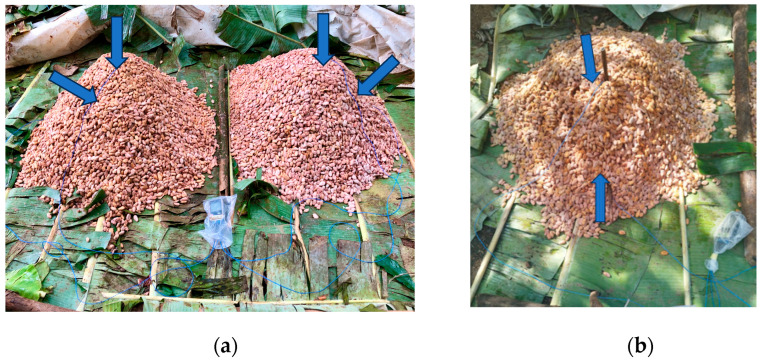
Positions of temperature probes in experimental runs A, B (**a**), and C (**b**), as indicated by blue arrows.

Temperature during fermentation was measured in 15 min intervals, using data loggers (testo 176T4, Testo AG, Mönchaltdorf, Switzerland). Two probes were installed per heap, one about 15–20 cm deep, in the middle, the second 15 cm from the side (in experimental runs A and B, from the side, Figure 2a; run C, from the front side, Figure 2b). In experimental run B, one data logger malfunctioned in run 2, so for variations PS1D0d6 and PS1D1d6, data were available from only one sensor. In run C, certain data points were missing due to malfunctioning data loggers. The affected variations and corresponding time periods comprised the following instances: PS1D0d5 (run 1, in one of the two heaps, entire fermentation), PS1D1d5 (run 3, in one of the two heaps, from 53.5 h before the end of fermentation), PS1D0d7 (run 1, in one of the two heaps, 0–119.75 h) and PS1D1d7 (run 2, both heaps, from 75 h to the end of fermentation; in run 3, one of the two heaps 53.75–117.5 h). Mean values from all measured data points were used for the evaluation (*n* = 8 for runs A and B and *n* = 12 for run C).

#### 2.3.3. Sugar and Organic Acid Analysis by HPLC, during Fermentation and of the Dried Beans

Sugars (sucrose, glucose and fructose) and organic acids (citric, lactic and acetic acids) in cotyledons were determined on the first (d0) and last days of fermentation (fEnd), as well as in the dried beans (dried). Samples from each heap were prepared according to the procedure in Romanens et al. [39,40]. High-performance liquid chromatography (HPLC) was performed on an HPLC system (Agilent 1260, Santa Clara, CA, USA). A ROA–Organic Acid H+ column was used for sucrose, glucose, citric acid, lactic acid, acetic acid and Rezex RPM monosaccharides for fructose. A refractive index (RI) detector (1260 RID G1362A, Agilent, Santa Clara, CA, USA, temperature set to 50 °C) was utilized for sugars, and a diode array detector (DAD) (1260 RID G4212B, Agilent, Santa Clara, CA, USA, wavelength set to 210 nm) for organic acid analyses [17]. Results were expressed as mg/g dry matter.

#### 2.3.4. Assessing the Viability of the Cocoa Embryo after Fermentation, and Cut-Tests of Dried Beans

**Figure 3:**
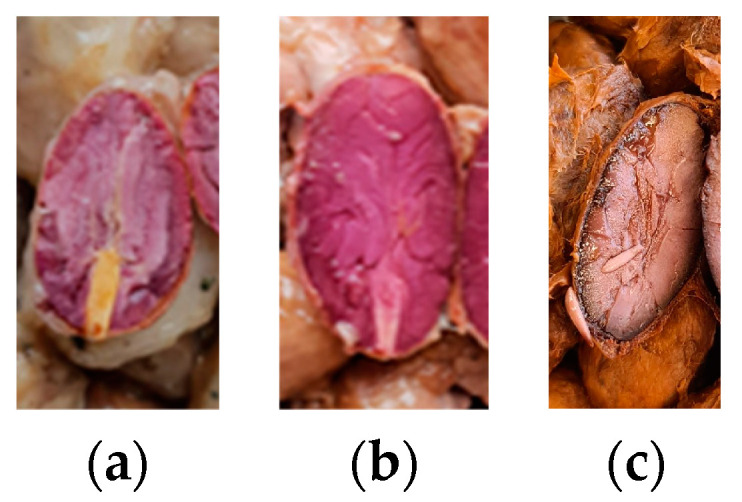
Exemplary images of embryo viability, interpreted as being active (**a**) and inactivated (**b**), (**c**) embryos.

After fermentation, 25 or 50 beans (50 in runs A and B, and 25 in C) from each heap, per run, were cut lengthwise to evaluate the embryo viability according to its color (interpreting active as white/beige and inactivate as violet/brown) (Figure 3). A cut-test of dried beans was performed as described in Streule et al., using 3 × 100 beans per heap [41].

#### 2.3.5. Fermentation Index (FI) of the Dried Beans

The fermentation index was determined following the method of Gourieva and Tserrevitinov [42], with minor modifications. Frozen cocoa nibs were milled for 30 s with an analytical mill (IKA, München, Germany) and 250 mg of milled nibs per heap were homogenized with 25 mL methanol:HCl (97:3 (*v*/*v*)) solution (methanol was supplied by VWR International GmbH, Dietikon, Switzerland and HCl by Carl Roth AG, Arlesheim, Switzerland). After storage for 17 h at 5 °C, the homogenate was filtered (Whatman filter paper). The absorbance was read three times at wavelengths of 460 nm and 530 nm (Synergy HTX, BioTek Instruments, Winooski, VT, USA), and the ratio of the values at 460 nm and 530 nm was calculated as FI.

### 2.4. Production and Analyses of Cocoa Liquor

#### 2.4.1. Production of Cocoa Liquor

Liquor was produced by combining independent runs and dependent repetitions of all variations. The cocoa beans were roasted for 25 min at 130 °C (Kompaktbackofen H5081-60 BP, Miele, Germany). Following roasting, beans were peeled using breaker and widower machines (Cocoa Breaker, Capco Test equipment, UK and Cocoa Widower Large, Capco Test equipment, UK). Cocoa liquor was produced in a Thermomix^®^ TM6 (Vorwerk, Wuppertal, Germany) according to the following protocol: mixing at 90 °C for 1 min at level 10, followed by 1 min at level 5, then 1 min at 100 °C at level 5, and finally, 3 min at 50 °C at level 5. The cocoa liquor was milled twice using a three-rolling mill (SDY, Bühler, Uzwil, Switzerland), with roll temperatures set at 35 °C, 44 °C and 10 °C; roll pressure at 19 bar; and blade pressure at 6 bar. The cocoa liquor was then further analyzed (Section 2.4.2 and Section 2.4.3).

All cocoa liquors from experimental runs A and B were subjected to sensory screening. Those samples whose sensory quality had dropped sharply due to extremely strong bitterness and astringency (samples with a fermentation period of less than 5 days) were not considered for further sensory and chemical characterizations.

Therefore, the cocoa liquors from samples A_PS1D0d5, A_PS1D1d5, B_PS1D0d6, B_PS1D1d6 and B_PS3D1d6 were subjected to sensory evaluation and chemical analyses. In order to confirm the observations made at the chemical level for runs A and B, targeted analyses of selected key compounds were conducted on the liquor samples from run C.

#### 2.4.2. Sensory Analysis

A consensus profile according to DIN EN ISO 13299 [43] was applied for the sensory characterization of the test samples. Eight trained panelists from the ZHAW Cocoa and Chocolate Panel evaluated the five selected cocoa liquor samples (Section 2.4.1) on three sensory categories (basic taste, trigeminal sensation and retronasal aroma), comprising a total of 11 attributes (Table 2). The panelists were informed about the research project’s objectives prior to the study and recruited on a voluntary basis. The study did not require formal ethical approval by the ethics committee of the canton of Zurich, Switzerland, as this study was not performing medical research, and therefore did not involve patients, but only healthy panelists. Several training sessions were conducted prior to the consensus-profiling to develop the sensory vocabulary, based on prior experiences of the panel; to define appropriate reference material (natural and aroma substances) for each tested attribute; and to train the judges on the different intensities of the selected attributes. The sensory attributes were individually evaluated on an 11-point categorical scale from 0 (=not perceivable) to 10 (=very strong). In a second step, and based on the individual results, the panel started a group discussion in which they developed the final consensus value for each attribute in each sample. All judges evaluated the samples in the same order in order to have a consensus discussion after each tested sample. To avoid a carry-over effect, warm water (40 °C) was used for neutralization between each sample.

All cocoa liquor samples were portioned into individual plastic jars (food grade), closed with a lid and stored in a climate-controlled cabinet at 15 °C. All samples were labelled with a three-digit random code according to the experimental design. Prior to each tasting, the samples were melted at 45 °C in a heating cabinet [9].

#### 2.4.3. Analysis of Selected Key-Aroma Compounds and Tastants in the Cocoa Liquor

##### Chemicals and Reagents

Chemicals used for sample preparation, extraction and quantitation of selected polyphenols and alkaloids:

Acetone (LC-MS grade) and water (LC-MS grade) were supplied by Carl Roth AG (Arlesheim, Switzerland). Acetonitrile and methanol (LC-MS grade) were supplied by Honeywell Deutschland Holding GmbH (Offenbach, Germany). Formic acid, theobromine and caffeine were purchased from Sigma-Aldrich (Merck AG, Zug, Switzerland). The n-Hexane was supplied by VWR International GmbH (Dietikon, Switzerland). Catechin, procyanidine B2 and cinnamtannin A2 were supplied by Phytolab GmbH & Co. KG (Vestenbergsgreuth, Germany). Epicatechin and procyanidine C1 were purchased from Extrasynthese (Genay, France).

Chemicals used for sample preparation, extraction and quantitation of selected aroma compounds:

Diethylether (99.5% Rotipuran) and anhydrous sodium sulfate (≥99%, p.a.) were supplied by Carl Roth AG (Arlesheim, Switzerland).

The stable isotopically substituted odorants 2-(^2^H_3_)methylbutanal, 3-methyl(3,5-^2^H_2_)butanal, ethyl 2-(^2^H_3_)methylbutanoate, ethyl 3-(^2^H_3_)methyl(2,2,3,4,4,4-^2^H_6_)butanoate, (^2^H_6_)dimethyl trisulfide, 2-(^2^H_5_)ethyl-3,6-dimethylpyrazine, 2-(^2^H_3_)methyl-3,5-dimethylpyrazine, 2-methyl-3-((^2^H_3_)methyldithio)furan, 3-(^2^H_3_)methyl-(2,2,3,4,4,4-^2^H_6_)butanoic acid, ethyl (^2^H_5_)phenylacetate, 3-methylbutyl-(^13^C_2_)acetate, 2-(^2^H_5_)phenylethanol, 2-(^2^H_3_)methoxyphenol, 4-methyl(2,6-^2^H_2_)phenol, 4-hydroxy-2-methyl-5-(^13^C)methyl(5-^13^C)furan-3(2H)-one, 3-hydroxy-4-methyl-5-(^13^C)methyl(5-^13^C)furan-2(5H)-one, 5-(1,2-^2^H_2_)hexyloxolan-2-one, 5-(4,4,5,5-^2^H_2_)pentyloxolan-2-one, 3(^2^H_3_)methyl-7-methyl-(4,4-^2^H_2_)octa-1,6-dien-3-ol, phenyl(^13^C_2_)acetic acid and phenyl(^13^C_2_)acetaldehyde were supplied by AromaLAB GmbH, Martinsried, Germany; (^13^C_2_)acetic acid was supplied by Merck KGaA, Darmstadt, Germany.

The reference odorants 2-methylbutanal, 3-methylbutanal, ethyl 2-methylbutanoate, ethyl 3-methylbutanoate, dimethyl trisulfide, 2-ethyl-3,(5 or 6)dimethylpyrazine, 2,3,5-trimethylpyrazine, 2-methyl-3-(methyldithio)furan, 2-methylbutanoic acid, 3-methylbutanoic acid, ethyl phenylacetate, 3-methylbutyl acetate, 2-phenylethanol, 2-methoxyphenol, 4-methylphenol, 4-hydroxy-2,5-dimethylfuran-3(2H)-one, 3-hydroxy-4,5-dimethylfuran-2(5H)-one, 6-pentyl-5,6-dihydro-2H-pyran-2one (δ-decenolactone), 5-pentyloxalan-2-one (γ-nonalactone), 3,7-dimethylocta-1,6-dien-3-ol (linalool), phenylacetic acid, phenylacetaldehyde and acetic acid were purchased from Merck KGaA.

##### Measurement of Selected Key Tastants

Extraction for analysis of selected polyphenols and alkaloids

Prior to extraction, the water and fat content levels of the liquors were measured (Supplementary Material, Figure S1). Extracts used for the quantification of single polyphenols and alkaloids by HPLC-MS were prepared as described by Schlüter et al. [44], with minor modifications. Briefly, the cocoa liquors were defatted as described by Schlüter et al. [44] before being extracted. In total, 0.5 g of defatted cocoa liquors was extracted three times with 2 mL of acetone/water (50:50, *v*/*v*) at 50 °C for 8 min using a heated laboratory shaker (Hettich AG, Tuttlingen, Germany) and subsequently centrifuged at 2880× *g* for 5 min (Type 5810, Vaudaux-Eppendorf AG, Schönenbuch, Switzerland). After centrifugation, supernatants were combined and filtered through a syringe filter (RC membrane, 0.2 μm, Phenomenex Helvetia GmbH, Basel, Switzerland) into HPLC glass vials, which were then sealed and stored at −20 °C until further analysis. All extractions were conducted in triplicate.

Quantitation of selected polyphenols and alkaloids by HPLC-UV-MS

For the quantification of the selected polyphenols, the extracts were diluted 1:2 prior to analysis with a solution containing 50% (*v*/*v*) acetonitrile in water. The HPLC-UV-MS analysis was conducted as described by Schlüter et al. [44], with modifications. HPLC−UV-mass spectrometry (MS) analysis was conducted on a system consisting of an Agilent 1290 Infinity II chromatographic system coupled to an Agilent 6530 Q-TOF mass spectrophotometer. The separation of analytes was performed using an Agilent Poroshell 120 EC-C18 (2.1 × 150 mm, 2.7 μm) column preceded by a guard column (Agilent Poroshell 120 EC-18, 2.1 × 5 mm, 2.7 μm). The flow rate was set to 0.7 mL/min, and the column temperature was set at 35 °C. The two elution mobile phases were made up of water +0.1% formic acid (FA) (mobile phase A) and acetonitrile +0.1% FA. HPLC gradient was as described in the following: 0−3 min, 5% B; 5–10 min, 9% B; 12–19 min, 18% B; 21 min, 30% B, 22−30 min, 100% B; 30.10−37 min, 5% B. The injection volume was 2 μL. UV spectra were recorded at 275 nm. The selected polyphenols were analyzed by mass spectrometry using an Agilent 6530 Q-TOF instrument in negative ionization mode (ESI−), in the spectral range of 100−1700 Da. Nitrogen served as the nebulizer and collision gas. The MS parameters were as follows: gas temperature, 350 °C; drying gas, 10 L/min; nebulizer, 40 psi; sheath gas temperature, 350 °C; sheath gas flow, 11 L/min; and capillary voltage, 4000 V. For the analysis of the alkaloid theobromine the acetone/water extracts were diluted 1:50 with a 50% (*v*/*v*) acetonitrile-in-water solution. For the analysis of caffeine, the acetone/water extracts were diluted 1:2 with the 50% (*v*/*v*) acetonitrile-in-water solution. The HPLC parameters were identical, but the detection was made using a UV detector with wavelength set at 275 nm. 

Pure substances were used for the determination of retention times and for the preparation of calibration solutions (Table 3). The contents of individual substances were calculated using linear regression. The results are expressed as g or mg/kg ffdm (fat-free dry matter).

##### Measurement of Selected Key Aroma Compounds

Sample Work-Up

The extracts were prepared as described by Ullrich et al. [37]. The cocoa liquor (1−20 g) was manually broken into pieces. Extraction was performed by stirring the cocoa liquor with an adequate amount of water (15–50 mL) and solvent (45–150 mL of previously distilled diethyl ether). Stable isotopically substituted odorants were used as internal standards and added in the beginning of extraction in an amount that was expected for the target odorant in the sample (0.008–2727 μg). The mixture was stirred at room temperature for at least 12 h. The diethyl ether phase of the extract was separated by means of a separating funnel and a centrifuge (10 min, 11,000 rpm, 14,610× *g*) and subjected to SAFE distillation [45]. The thawed SAFE distillate was dried over anhydrous sodium sulfate and concentrated to a volume of 300 μL using a Vigreux column and, in the last step, a gentle stream of nitrogen. All extractions were conducted in triplicates.

Quantitation of odorants by Gas Chromatography–Mass Spectrometry (GC-MS)

Quantitation was performed as described by Ullrich et al. [37], with either a GC-MS (I) or a GC-GC-MS (II) system, depending on the target compound (details in Table S1). The GC-MS system consisted of a TRACE GC Ultra (Thermo Fisher Scientific, Reinach, Switzerland) coupled to a TSQ Quantum mass spectrometer (Thermo Fisher Scientific) operating in an EI mode. Volatiles were separated on a DB-FFAP column (30 m length, 0.25 mm inner diameter, 0.25 μm film thickness; Agilent Technologies, Basel, Switzerland) using a constant helium carrier flow of 2.5 mL/min. An autosampler injected 1 μL of cold sample directly on-column. The GC oven was tempered at 40 °C for 4 min, and then this was increased by 7 °C/min until 240 °C was reached. The MS transfer line was heated to 280 °C, the ion source temperature was 200 °C and the electron ionization energy was 70 eV. The GC-GC-MS system was the same as that previously described in [46]. Deviating parameters included an injection volume of 1 μL, a temperature of 240 °C for the transfer line between the two GCs, and the temperature and gas flow programs of both GCs. The oven temperature programs started with 40 °C for both columns, except for the measurements of 2-methylbutanal and 3-methylbutanal, with 30 °C as initial temperature for the second oven. The first oven was started after 4 min, with temperatures increasing 8 °C/min until 240 °C was reached. The second oven started heating 4 min after the ending time of the heart cut and was increased at 8 °C/min up to 240 °C and then increased to 50 °C/ min until 270 °C was reached. The end temperatures were held until the end of the run. The cut times were variable and depended on the retention times of the target compounds on the first column. A cold trap (Brechbühler AG, Schlieren, Switzerland) in the second oven was operated at −140 °C until 0.1 min after the cut, and was then rapidly heated up to 220 °C. The front inlet pressure was 180 kPa and the middle inlet pressure was 150 kPa. The middle inlet fed helium directly to the MCSS. The pressures were increased to 250 kPa for the middle inlet and 280 kPa for the front inlet 0.1 min after the heart cut. The MS in both systems was operated in the selected ion monitoring (SIM) mode, with individual quantifier ions for the analyte and the standard for each target compound. The odorant concentration in the sample was calculated by means of the peak areas of the analyte and the standard, the amount of the added standard, and the sample weight by employing calibration line equations. Mixtures of the analyte and the standard in at least five different ratios (5:1, 2:1, 1:1, 1:2 and 1:5) were measured with the above-described methods, and the peak area ratios were calculated to obtain a calibration line equation by linear regression. For each target compound, the quantifier ions, the calibration line equation and the quantitation system used are detailed in Table S1.

### 2.5. Statistics

In total, fermentation variations from experimental runs A and B were performed four times and six times in experimental run C (Table 1). For normally distributed data (determined using the Shapiro–Wilk test and/or a Q–Q plot), a one-way ANOVA, followed by post hoc (Tukey-HSD Test), was applied for each experimental run. For pairwise comparisons using samples with/without depulping, with 1- and 3-day pod storage, or experimental runs carried out in different years A/B (2022) and C (2023), a *t*-test with Bonferroni correction was applied.

Multivariate analysis using partial least squares (PLS) was applied to visualize relations and variations between the fermentation processes and the quality parameters of the cocoa beans (viability of embryo, cut-test, and fermentation index). Predictor and response variables were selected according to significant differences and tendencies between variations. Mean values of the repetitions were used. All these analyses were performed using RStudio (version 4.3.0).

Regarding sensory analysis, the consensus values as determined by group discussion were graphically presented in a bar chart created with Excel (Microsoft Office Excel 365 ProPlus). Principal component analysis (PCA) and hierarchical cluster analysis (HCA), with Euclidean distance as dissimilarity measure and unweighted pair–group average as an agglomeration method, was applied to the data set (consensus values) using XLSTAT Premium (version 2023.1.5).

To study the influences of depulping, pod storage, and fermentation on the key selected aroma and tastants compounds, a mixed-model ANOVA analysis was conducted by defining the experimental runs as a random factor and the depulping, the pod storage, and the fermentation as three different fixed factors, following the random effect mixed model (REML) and using XLSTAT. A heat map with the volatile and non-volatile concentrations was created using XLSTAT.

All the tests were performed at a significance level of α = 0.05. 

## 3. Results

### 3.1. Post-Harvesting Process Monitoring and Quality of Dried Cocoa Beans

#### 3.1.1. Pulp Content

**Figure 4:**
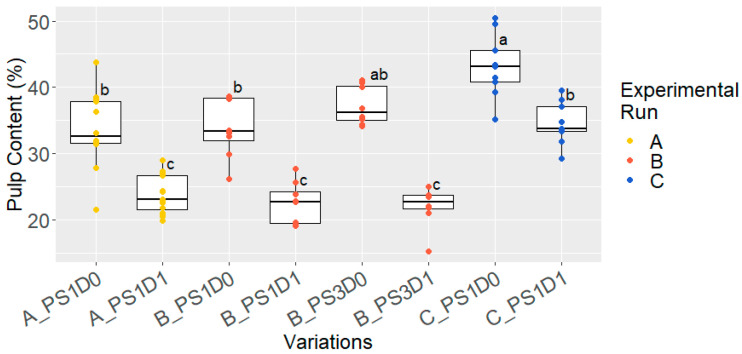
Pulp content (%) at start of fermentation, for variations with 1- or 3-day pod storage (PS1 and PS3, respectively) and without and with depulping (D0 and D1, respectively) for the three experimental runs, A (gold, *n* = 10 for D0 and *n* = 18 for D1), B (red, *n* = 8) and C (blue, *n* = 9). Variations differ significantly if they do not share a common letter.

At the start of fermentation, pulp content was measured from variations without (D0), and with, depulping (D1) (Figure 4). Throughout each experimental run, mechanically depulped cocoa samples exhibited significantly lower pulp-content levels compared to those that had not been depulped (*p* < 0.05). In experimental runs A and B, similar pulp content was measured: 35.3 ± 4.2% for D0, and 23.2 ± 3.3% for the D1 sample. In experimental run C, a higher overall pulp content was measured, with significantly more pulp present in the depulped (34.6 ± 3.2%) and non-depulped (43.2 ± 4.8%) samples compared to runs A and B (23.2 ± 3.3% and 35.3 ± 4.2%, respectively), as confirmed by a t-test (*p* < 0.05).

#### 3.1.2. Cotyledon pH during Fermentation and in Dried Beans

The pH determinations of the cotyledon at the start of the fermentation (d0) were similar in experimental runs A and B (6.54 ± 0.08), while significantly lower values (6.43 ± 0.05) were observed in run C (*p* < 0.05) (Table 4). Slightly elevated pH values in experimental run B were observed when pods were stored for 3 days. As expected, pH levels steadily declined during the course of fermentation across all variations. Trends emerged, particularly at d2, d3 and d4. In experimental run A, the variation without depulping (A_PS1D0d5) showed significantly higher pH values (6.48 ± 0.04) than those for the variation with depulping (pH 6.36 ± 0.04) (*p* = 0.00031) on d2, but values were significantly lower (*p* = 0.052) on d3 and d4, compared to samples with depulping. In experimental run B, samples with 3-day pod storage tended to exhibit lower pH levels on d2 and d3. Samples subjected to depulping in experimental run C displayed significantly lower pH values (pH 5.72 ± 0.30) than those without depulping (pH 6.05 ± 0.07) (*p* = 0.026), on d2. The pH values remained constant from d5 to d6 and d7 in all samples from experimental run B, as well as in samples fermented for 7 days in run C.

Across all variations it could be observed that the longer the fermentation duration, the lower the pH values at the end of the fermentations; this was particularly noticeable at points between 3 and 5 days. For fermentations lasting longer than 5 days, the pH remained similar or slightly increased, but the end values were always lower than at d0. 

In dried beans, pH values did not show significant differences (*p* > 0.05), yet the trend of slightly higher values in depulped samples remained consistent across all experimental runs. The only significant differences in dried samples were in B_PS1D0d6 vs B_PS3D1d6 (*p* = 0.048). Given the marginal *p*-value, this result should be interpreted with caution.

#### 3.1.3. Temperature Development in Fermentation Heaps

In all three experimental runs, a similar tendency in temperature development was observed when comparing samples with and without depulped beans (Table 5). The maximum temperatures in the initial hours of fermentation (59–60 h in experimental run A; 58–62 h in run B; 60–63 h in run C) were higher for the depulped samples, compared to the non-depulped samples, although the difference was not statistically significant (*p* > 0.05).

In experimental run A, the non-depulped samples (A_PS1D0d5) reached a temperature of 36.5 ± 3.1 °C, while the depulped samples (A_PS1D1d3, A_PS1D1d4, A_PS1D1d5) averaged 38.3 ± 3.7 °C (*p* > 0.05). In experimental run B, on d2, the non-depulped samples reached 37.1 ± 1.7 °C (B_PS1D0d6) and 40.7 ± 2.8 °C (B_PS3D0d6), whereas the depulped samples reached 39.0 ± 2.7 °C (B_PS1D1d6) and 42.0 ± 3.1 °C (B_PS3D1d6) (*p* > 0.05).

In experimental run C, significant temperature differences were only observed on d1. The maximal temperatures in the non-depulped beans were significantly lower, reaching 35.0 ± 2.5 °C (C_PS1D0d5) and 35.3 ± 1.9 °C (C_PS1D0d7), compared to the depulped samples, which had temperatures of 38.0 ± 2.5 °C (C_PS1D1d5) and 38.3 ± 3.0 °C (C_PS1D1d7) (*p* < 0.05).

Furthermore, in experimental run B, differences in temperatures were observed between samples with pod storage of 3 days (PS3) versus those with 1 day (PS1). Samples associated with pod storage of 3 days showed a tendency for higher temperatures from day 2 to day 4, but a significant difference was observed only on d4, with PS3 samples showing temperatures of 47.4 ± 1.6 °C (both B_PS3D0d6 and B_PS3D1d6), which was significantly higher, compared to 45.6 ± 2.3 °C for PS1 (both B_PS1D0d6 and B_PS1D1d6) (*p* = 0.019).

Maximal temperatures decreased slightly after day 4 (experimental run A) or day 5 (runs B and C) until the end of fermentation.

In general, the ambient temperatures during the day (6 a.m.–6 p.m.) of experimental runs A and B were slightly lower (26.7 ± 2.8 °C and 26.1 ± 4.1 °C, respectively) compared to run C (29.6 ± 3.6 °C).

#### 3.1.4. Sugars and Organic Acids during Fermentation and in the Dried Beans

**Figure 5:**
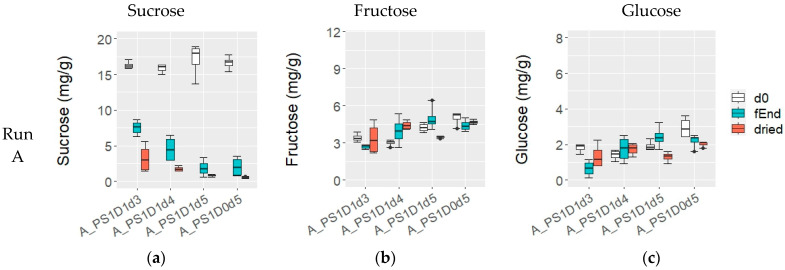

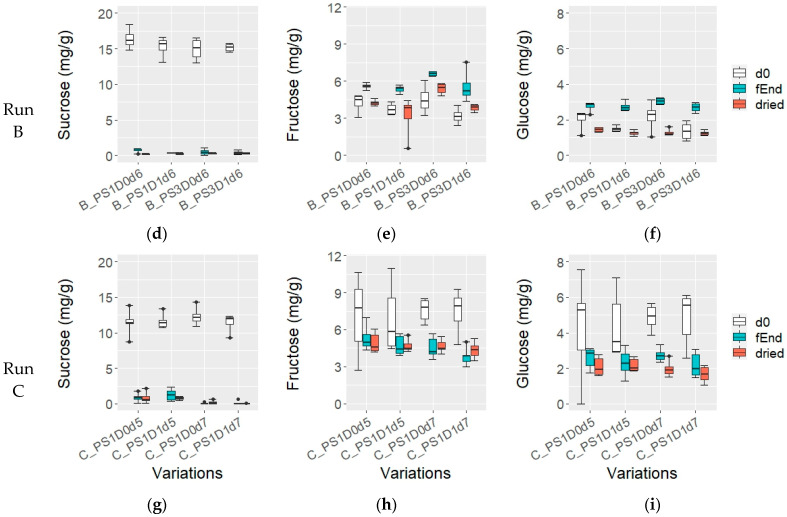
Sucrose (**a**,**d**,**g**), fructose (**b**,**e**,**h**), and glucose (**c**,**f**,**i**) (mg/g) at fermentation start (d0, white), fermentation end (fEnd, turquoise) and in dried beans (dried, red), in samples with 1 (PS1) or 3 (PS3) days pod storage, without (D0) or with (D1) depulping, and fermented for 3, 4, 5, 6 or 7 days (d) from experimental runs A ((**a**–**c**); *n* = 4), B ((**d**–**f**); *n* = 4) and C ((**g**–**i**); *n* = 6).

At the beginning of fermentation, the sucrose content was 16.0 ± 1.4 mg/g in experimental runs A and B, while in run C, it was significantly lower, at 11.7 ± 1.2 mg/g (*p* < 0.05) (Figure 5a,d,g). No notable differences were observed among the variations in terms of depulping, pod storage, or fermentation time. Throughout the fermentation process, the sucrose content decreased in all fermentations; however, samples with shorter fermentation times (A_PS1D1d3 and A_PS1D1d4) exhibited significantly higher sucrose levels (7.5 ± 1.1 mg/g and 4.5 ± 1.8 mg/g, respectively), compared to those fermented for 5 days (samples from run A, 2.0 ± 1.2 mg/g), 6 days (all samples from run B, 0.5 ± 0.3 mg/g) and 7 days (from run C, 0.1 ± 0.2 mg/g) (*p* < 0.05). Dried beans from experimental runs B and C showed sucrose levels similar to those at the end of fermentation. And in run A, shorter fermentations (A_PS1D1d3 and A_PS1D1d4) exhibited significantly higher final sucrose values (3.2 ± 2.0 mg/g and 1.7 ± 0.4 mg/g, respectively), compared to those fermented for 5 days (0.7 ± 0.2 mg/g) (*p* < 0.05).

At d0, fructose content was generally consistent across variations, although slightly lower values were observed in samples A_PS1D1d3 and A_PS1D1d4 compared to A_PS1D1d5 and A_PS1D0d5 in run A. Notably, fructose content at d0 was significantly higher in run C (7.3 ± 2.1 mg/g), compared to runs A and B together (3.9 ± 0.9 mg/g) (*p* < 0.05) (Figure 5b,e,h). Significantly higher fructose levels were detected at the end of fermentation in run A in samples fermented for 5 days compared to 3 days (4.7 ± 0.8 mg/g and 2.7 ± 0.2 mg/g, respectively; *p* < 0.05), while no significant differences were observed in run B (*p* > 0.05). In run C, fructose content was significantly lower in C_PS1D1d7 compared to C_PS1D0d5 (3.8 ± 0.7 mg/g and 5.3 ± 1.0 mg/g, respectively; *p* < 0.05). In dried beans, fructose content was similar across samples in runs A and B, averaging at 4.1 ± 1.0 mg/g (with a slight tendency in run B towards higher values in samples without depulping). Run C also showed similar fructose content (4.7 ± 0.6 mg/g), but it was significantly different from runs A and B (*p* = 0.015).

At the start of each experimental run, glucose levels were generally consistent across variations (Figure 5c,f,i), except for a significantly higher glucose level in the non-depulped sample A_PS1D0d5, compared to other variations within the same run (*p* = 0.01424). In run C, glucose content was significantly higher, at 4.6 ± 1.7 mg/g compared to 1.9 ± 0.7 mg/g in runs A and B (*p* < 0.05). At the end of fermentation in experimental run A, glucose content was significantly lower in A_PS1D1d3 (0.7 ± 0.5 mg/g), compared to samples fermented for 4 (1.7 ± 0.8 mg/g) or 5 (2.3 ± 0.5 mg/g) days (*p* < 0.05). No differences were observed among samples in run B (2.8 ± 0.3 mg/g) or run C (2.4 ± 0.6 mg/g) in terms of depulping, pod storage or fermentation time. In dried samples, glucose levels did not significantly differ among samples (*p* > 0.05), except for a difference between experimental runs A and B (1.5 ± 0.4 mg/g) compared to run C (2.0 ± 0.5 mg/g) (*p* < 0.05).

**Figure 6:**
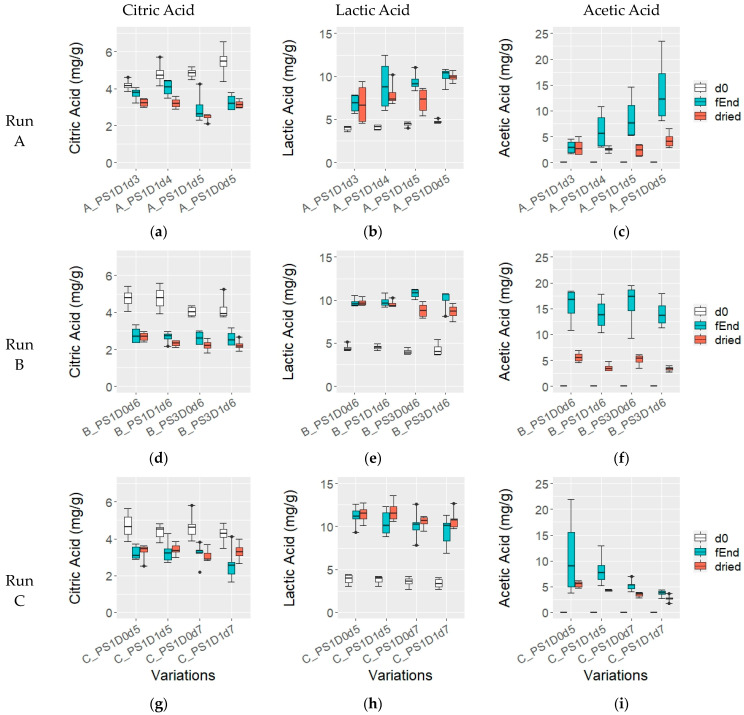
Citric acid (**a**,**d**,**g**), lactic acid (**b**,**e**,**h**), and acetic acid (**c**,**f**,**i**) (mg/g) at fermentation start (d0, white), fermentation end (fEnd, turquoise) and in dried beans (dried, red), in samples with 1 (PS1) or 3 (PS3) days pod storage, without (D0) or with (D1) depulping, and fermented for 3, 4, 5, 6 or 7 days (d), from experimental runs A ((**a**–**c**); *n* = 4), B ((**d**–**f**); *n* = 4) and C ((**g**–**i**); *n* = 6).

The citric acid (CA) (Figure 6a,g,d) content remained stable at 4.6 ± 0.6 mg/g across all experimental runs (*p* > 0.05). However, during fermentation, CA levels decreased consistently. In experimental runs B and C, CA decreased to 2.7 ± 0.4 mg/g and 3.1 ± 0.6 mg/g, respectively until the end of fermentation. In experimental run A, samples fermented for 5 days had significantly lower CA compared to 3 and 4 days (3.1 ± 0.7 mg/g and 3.9 ± 0.4 mg/g, respectively; *p* = 0.016). In dried beans, CA content was significantly lower in sample A_PS1D1d5 (2.5 ± 0.2 mg/g) compared to the other samples with different fermentation durations (3.2 ± 0.2 mg/g) from run A (*p* < 0.05). 

At the beginning of fermentation, lactic acid (LA) levels were 4.3 ± 0.5 mg/g in experimental runs A and B, and significantly lower at 3.6 ± 0.5 mg/g in run C (*p* < 0.05) (Figure 6b,e,h), and levels increased over the course of fermentation. In run A, samples fermented for 5 days had more LA compared to 3 days (6.8 ± 1.1 mg/g and 9.7 ± 01.1 mg/g, respectively; *p* = 0.0014). After drying, LA remained similar in all samples except for A_PS1D1d5, which had lower levels than the sample at the end of fermentation and significantly less than A_PS1D0d5 (7.2 ± 1.6 mg/g and 9.9 ± 0.6 mg/g, respectively; *p* = 0.016). In run B, LA was slightly higher in the sample with 3-day pod storage compared to the 1-day at the end of fermentation (*p* = 0.16), and significantly lower in dried beans with 3-day pod storage compared to 1-day (8.7 ± 0.8 mg/g and 9.7 ± 0.4 mg/g, respectively; *p* = 0.016). In run C, LA levels remained constant at the end of fermentation (10.3 ± 1.5 mg/g) and after drying (11.1 ± 1.0 mg/g) across all samples.

In experimental run A (Figure 6c), the acetic acid (AA) content increased across the variations: starting from 0 mg/g at the fermentation start, it reached 3.1 ± 1.4 mg/g in A_PS1D1d3, 7.5 ± 4.0 mg/g in A_PS1D1d4 and A_PS1D1d5, and 13.9 ± 3.3 mg/g in A_PS1D0d5. The AA content in PS1D0d5 was significantly higher than in A_PS1D1d3 (*p* < 0.05). However, in dried beans, AA content was similar across all samples, with 3.1 ± 1.4 mg/g. In run B (Figure 6f), AA content increased until the end of fermentation to 4.4 ± 1.3 mg/g with no significant differences among samples. Dried samples showed significantly higher AA in non-depulped (5.3 ± 1.1 mg/g) rather than depulped (3.5 ± 0.6 mg/g) samples (*p* = 0.00077). In experimental run C (Figure 6i), AA increased until the end of fermentation, with lower amounts in depulped samples compared to levels in non-depulped samples fermented for 7 days (3.7 ± 0.6 mg/g and 5.3 ± 1.0 mg/g, respectively; *p* = 0.011). Samples fermented for 7 days had significantly less AA than those fermented for 5 days (4.5 ± 1.1 mg/g and 9.5 ± 5.5 mg/g, respectively; *p* = 0.0049). In dried samples, AA content mirrored levels at the end of fermentation, with significantly less AA in samples fermented for 7 days compared to 5 days (3.1 ± 0.7 mg/g and 4.9 ± 0.8 mg/g, respectively; *p* < 0.05), and significantly less in depulped (C_PS1D1d5 and C_PS1D1d7, 3.5 ± 0.9 mg/g) than in non-depulped (C_PS1D0d5 and C_PS1D0d7, 4.5 ± 1.2 mg/g) samples (*p* = 0.03).

#### 3.1.5. Viability of Cocoa Embryo after Fermentation and Cut-Test of Dried Beans

**Figure 7:**
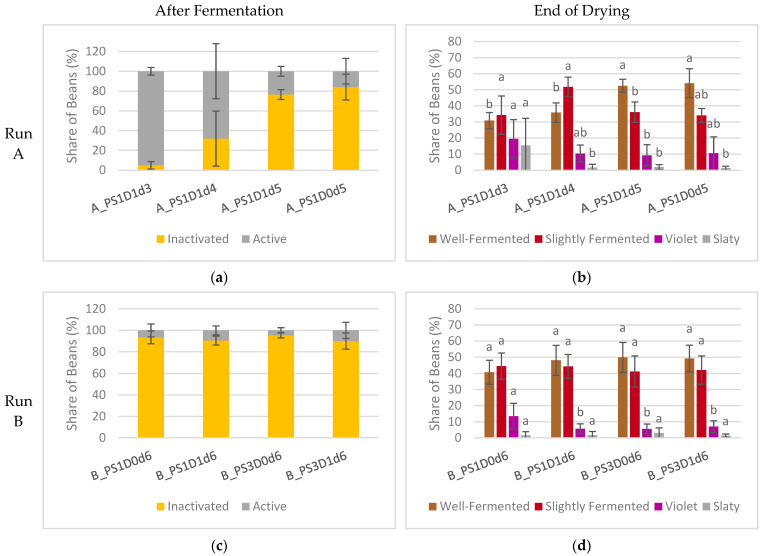

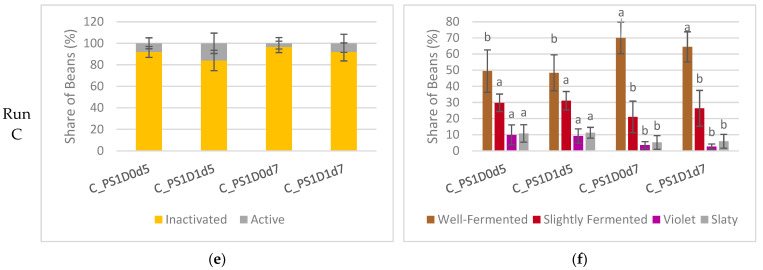
(**a**,**c**,**e**) Viability of cocoa embryo after fermentation in samples with 1 (PS1) or 3 (PS3) days pod storage, without (D0) or with (D1) depulping, and fermented for 3, 4, 5, 6 or 7 days (d), from experimental runs A ((**a**), *n* = 4), B ((**c**), *n* = 4) and C ((**e**), *n* = 6). The embryos were classified as inactivated (yellow) or active (grey). (**b**,**d**,**f**) Cut-test at the end of drying in the same samples from experimental runs A ((**b**), *n* = 12), B ((**d**), *n* = 12) and C ((**f**), *n* = 18). The beans were classified as well-fermented (brown), slightly fermented (red), violet (violet), or slaty (grey). Bars of the same color representing the same classification differ significantly if they do not share a common letter.

With a prolonged fermentation duration, a notable increase in the occurrence of inactivated embryos was evident in experimental run A (Figure 7a). Samples fermented for 5 days exhibited a significantly higher proportion of inactivated embryos, at 80 ± 10%, compared to those fermented for 3 or 4 days, which showed only 19 ± 23% inactivated embryos (A_PS1D1d3, A_PS1D1d4) (*p* < 0.05). There were no differences observed between beans subjected to depulping and those not subjected. In experimental run B (Figure 7c), the share of inactivated embryos remained consistent across all samples, at 92 ± 5%, irrespective of pod storage or depulping (*p* > 0.05). Similarly, in experimental run C (Figure 7e), no significant differences were noted in terms of either duration or depulping (91 ± 8% overall). A trend towards a lower occurrence of inactivated embryos was observed when depulped beans were fermented for 5 days, compared to non-depulped beans (*p* = 0.098).

In experimental run A, the cut-test of dried beans (Figure 7b) revealed significant differences among samples with varying fermentation durations. Specifically, samples fermented for 5 days (PS1D1d5, PS1D0d5) exhibited a significantly higher share of well-fermented beans (53 ± 7% and 33 ± 6%, respectively, *p* < 0.05) and a lower proportion of slightly fermented beans, compared to those fermented for 3 or 4 days (PS1D1d3, PS1D1d4). Furthermore, sample PS1D1d3 demonstrated significantly more slaty beans (15 ± 17% for PS1D1d3 and 1.6 ± 1.6% for the others from the same run, *p* < 0.05) and a higher incidence of violet beans than the other samples. Notably, no significant differences were observed between samples with and without depulping. 

In experimental run B, no significant differences were observed among the four samples except for a higher incidence of violet beans in sample B_PS1D0d6 (14 ± 8%) compared to the others (6 ± 3%) (*p* < 0.05) (Figure 7d). However, a t-test indicated a significant difference in the percentages of well-fermented beans between the samples. Samples subjected to 3 days of pod storage (B_PS3D0d6, B_PS3D1d6) showed a higher percentage (50 ± 9%) of well-fermented beans compared to those stored for only 1 day (44 ± 9%) (*p* = 0.048). 

In experimental run C, differences in samples fermented for different durations were detected (Figure 7f). Depulping did not show significant differences (*p* > 0.05). Samples fermented for 7 days (C_PS1D0d7, C_PS1D1d7) resulted in significantly more well-fermented beans (49 ± 12% and 67 ± 10%, respectively) and significantly less violet (vs 3 ± 2% and 10 ± 5%, respectively) and slaty beans (6 ± 4% and 11 ± 4%, respectively) than found in samples fermented for 5 days (C_PS1D0d5, C_PS1D1d5) (*p* < 0.05).

#### 3.1.6. Fermentation Index of Dried Beans

**Figure 8:**
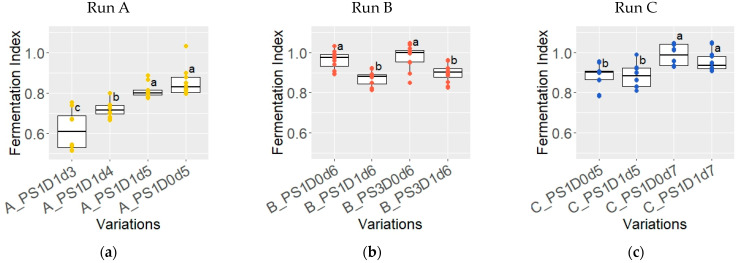
Fermentation index for dried beans from samples with 1 (PS1) or 3 (PS3) days pod storage, without (D0) or with (D1) depulping, and fermented for 3, 4, 5, 6 or 7 days (d), from experimental run A in yellow ((**a**), *n* = 12), B in red ((**b**), *n* = 12), and C in blue ((**c**), *n* = 18). Variations differ significantly if they do not share a common letter.

The fermentation index (FI) exhibited notable variations across different fermentation durations in experimental runs A and C (Figure 8a,c). In experimental run A, the FI of samples fermented for 3 days (A_PS1D1d3) was 0.62 ± 0.10, significantly lower than those fermented for 5 days (A_PS1D0d5 and A_PS1D1d5), with a FI of 0.83 ± 0.06 (*p* < 0.05). A similar trend was observed in experimental run C, in which, regardless of depulping, samples fermented for 5 days (C_PS1D0d5 and C_PS1D1d5) had significantly lower FI values compared to those fermented for 7 days (0.89 ± 0.06 and 0.97 ± 0.06, respectively, *p* < 0.05). Furthermore, the FI from variations A_PS1D0d5 and A_ PS1D1d5 were significantly lower in experimental run A compared to C (*p* = 0.00045). In experimental run B (Figure 8b), no significant differences were found between samples stored for 1 day versus 3 days (*p* > 0.05). However, depulped samples exhibited significantly lower FI values, compared to non-depulped samples (0.88 ± 0.04 and 0.97 ± 0.05, respectively, *p* < 0.05).

### 3.2. Results of Cocoa Liquors Analyses

Cocoa liquors from samples A_PS1D0d5, A_PS1D1d5, B_PS1D0d6, B_PS1D1d6 and B_PS3D1d6 were analyzed to evaluate the impacts of depulping, pod storage and fermentation time on their sensory profile and their chemical composition.

#### 3.2.1. Sensory Evaluation

**Figure 9:**
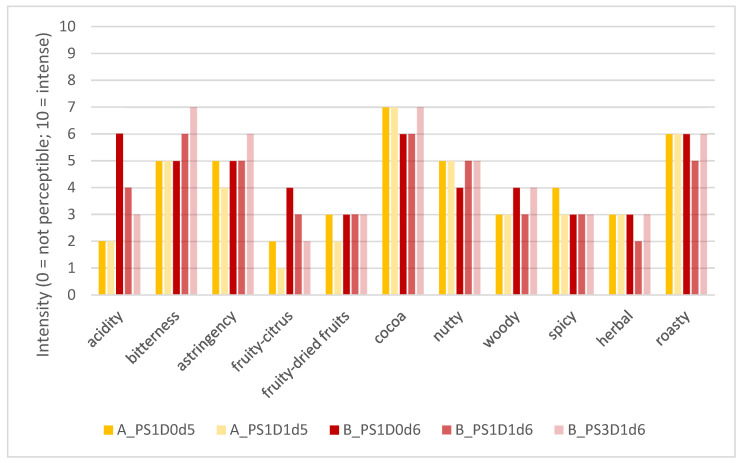
Consensus profiles of the test samples (cocoa liquors) with 1 (PS1) or 3 (PS3) days pod storage, without (D0) or with (D1) depulping, and after fermentation for 5 or 6 days (d), from experimental run A in yellow and experimental run B in red.

Figure 9 represents the product profiles, i.e., consensus values, of the evaluated cocoa liquors, considering the different attributes in the main categories of basic taste, trigeminal sensation, and aroma (retronasal).

All five cocoa liquor samples could be characterized based on the defined attributes. Sensory differences could be observed, mainly for taste characteristics like acidity and bitterness, and for the aroma attribute fruity-citrus. For sample B_PS3D1d6, judges perceived some earthy/mushroom-like notes (qualitative remark and no quantification of these attributes). Sample A_PS1D0d5 was characterized by low acidity, medium bitterness and astringency, with intense cocoa, medium nutty and some spicy notes. Sample A_PS1D1d5 was characterized by low acidity and few fruity notes but intense cocoa and medium nutty notes, whereas sample B_PS1D0d6 was characterized by intense acidity and cocoa, and medium fruity-citrus notes. Sample B_PS1D1d6 revealed medium acidity, medium-high bitterness and astringency, as well as cocoa notes and slightly fruity notes. The sample B_PS3D1d6 showed low acidity but intense bitterness and astringency. The aroma was characterized by cocoa, nutty, and some woody notes. The sensory profiles revealed that the sample obtained from pods with a pod storage time of 1 day (B_PS1D1d6) was characterized by a more intense acidity, less intense bitterness and astringency, and no earthy/mushroom-like notes, compared to the sample obtained from pods with the longer pod storage time of 3 days (B_PS3D1d6). Samples obtained from shorter fermentation times (5 versus 6 days) showed less intense acidity, fewer fruity-citrus notes, and slightly more intense cocoa notes. Samples obtained from depulped beans showed slightly fewer fruity-citrus notes compared to non-depulped beans.

#### 3.2.2. Chemical Analyses of Cocoa Liquor

##### Quantification of Selected Key Cocoa Tastants

The results of the quantifications of some selected key cocoa tastants in the liquors are shown in Table 6. To assess the impacts of the depulping and of the pod storage on those compounds, a mixed-model ANOVA analysis was conducted. The experimental runs were defined as a random factor, while the depulping and the pod storage were treated as two different fixed factors, following the random effect mixed model (REML).

Key cocoa tastants theobromine, caffeine, catechin, epicatechin, procyanidine B2, procyanidine C1 and cinnamtannin A2 were quantified in the cocoa liquors. The average concentrations of the selected compounds, as well as the results of the statistical analysis investigating the impact of depulping and pod storage on the compounds’ concentrations, are summarized in Table 6. 

It can be observed that the depulping of the cocoa beans before the fermentation had no influence on the content of the key tastants in the liquors, with the exception of catechin, where a significant difference was observed. A significant difference was found between the catechin concentrations in the samples of run A (A_PS1D1d5 vs A_PS1D0d5, ANOVA, *p* < 0.05), with the depulped samples showing significantly more catechin than the non-depulped samples. This difference was not observed in runs B and C.

The pod storage showed significant impacts on the selected taste-active compounds measured in the cocoa liquors (Table 6). The two alkaloids, theobromine and caffeine, remained stable during the duration of the pod storage. However, the contents of the measured polyphenols were significantly different in the beans from pods stored for 3 days in comparison to those from the pods stored for 1 day. Overall, fewer flavanols with polymerization degrees of 1 to 4 were found in the samples from 3-day pod storage.

##### Selected Key Aroma Compounds

Selected key odorants responsible for the characteristic aroma of cocoa were quantified in cocoa liquor samples, using GC-MS or GC/GC-MS (Table 7). As for the key tastants, the impacts of the depulping and of the pod storage on those compounds were investigated using a mixed-model ANOVA analysis. The *p*-values resulting from this statistical analysis are also given in Table 7.

The first observation is that the content levels of key aroma compounds found in experimental runs A and B (beans harvested in 2022) were lower than the ones found in the liquors produced from the beans of experimental run C (beans harvested in 2023) (Table 7). Furthermore, the depulping and the pod storage time had a significant impact on more than half of the selected odor-active compounds analyzed in the cocoa liquors (Table 7). 

The data in Table 7 show that depulping of cocoa beans leads to significantly lower contents of acids (acetic acid, 2- and 3-methylbutanoic acid), esters (3-methylbutyl acetate, ethyl phenylacetate), pyrazines (2,3,5-trimethylpyrazine, 2-ethyl-3,5-dimethylpyrazine) and sotolone. Depulped samples contained significantly higher 2-phenylethanol, 4-methylphenol, phenylacetaldehyde and linalool, compared to non-depulped samples. Finally, the impacts of depulping on gamma-nonalactone and dimethyl trisulfide cannot be clearly explained, as lower contents were found in the depulped samples for run A and run B, but higher contents were found in depulped samples of run C. The depulping showed no impact on other key aroma compounds analyzed.

The longer pod storage of 3 days resulted in higher concentrations of phenylacetaldehyde, pyrazines, linalool and 2-methoxyphenol, and lower amounts of acids (acetic acid, 2- and 3-methylbutanoic acid), esters (3-methylbutylacetate, ethyl phenylacetate, ethyl 2- and 3-methylbutanoate), 2-phenylethanol, sotolone and gamma-nonalactone, in comparison with the 1-day pod storage, in cocoa liquors made from the respective beans. 

### 3.3. Multivariate Analyses

#### 3.3.1. Effects of Post-Harvesting Parameters on Selected Quality Parameters of Dried Beans

**Figure 10:**
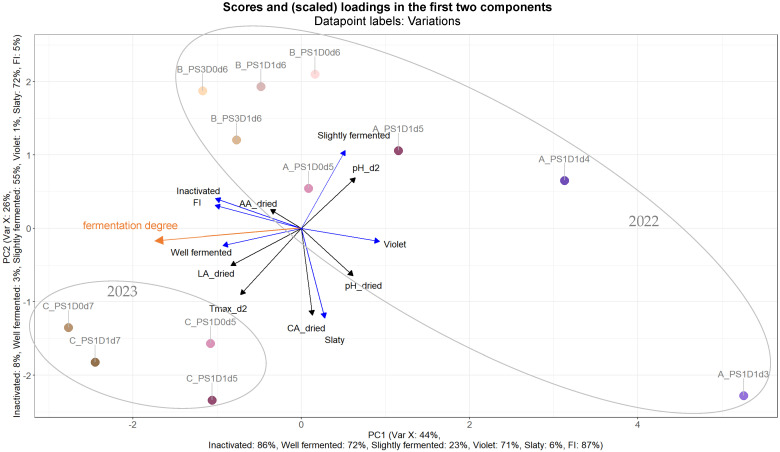
PLS (partial least squares) of predictors in black: pH value at d2 (pH_d2), pH value of dried beans (pH_dried), maximum temperature at d2 (Tmax_d2), citric acid of dried beans (CA_dried), lactic acid of dried beans (LA_dried), and acetic acid of dried beans (AA_dried). Response variables in blue: inactivated embryo (inactivated) and well fermented, slightly fermented, violet, and slaty beans; fermentation index (FI) of samples with 1 (PS1) or 3 (PS3) days pod storage; without (D0) or with (D1) depulping; and fermented for 3, 4, 5, 6 or 7 days (d) (direction of fermentation degree manually inserted in orange) from experimental runs A, B (carried out in 2022) and C (carried out in 2023).

A PLS (partial least squares) analysis was constructed to illustrate the influences of the fermentation variations, as to differences in pod storage duration, depulping and fermentation time, on fermentation and on cocoa bean quality (viability of embryo at the end of fermentation, fermentation index, and cut-test). Each data point (Figure 10) was annotated with its corresponding experimental run and variation. Groups were manually delineated to indicate whether the experiments were conducted in 2022 (runs A and B) or in 2023 (run C), a designation which is clearly visible in the PLS. With regards to variance, 44% and 26% can be attributed to PC1 and PC2, respectively. Notably, the variations are arranged in accordance with fermentation time, with those on the right side of the PLS plot representing shorter fermentation times (A_PS1D1d3 and A_PS1D1d4) and those on the left indicating longer durations, up to 7 days. Further, the direction of fermentation degree (manually inserted) is visible from the right (more violet/slaty beans) to the left (more well-fermented beans) sides of the PLS. Moreover, some variations without depulping (D0) tend to cluster slightly more towards the left side of the plot, such as A_PS1D0d5, B_PS3D0d6, and C_PS1D0d7. Additionally, within run B, variations with 3-day pod storage exhibit a tendency to lean more leftward compared to those with only 1-day pod storage.

#### 3.3.2. Principal Component Analysis for Sensory Analysis and Heat-Map Clustering for Chemical Analyses

**Figure 11:**
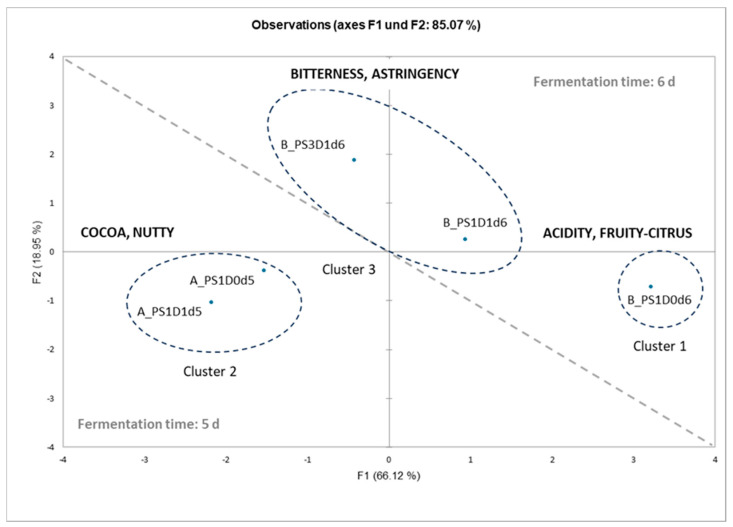
Principal component analysis and results from cluster analysis of the test samples (cocoa liquors) with 1 (PS1) or 3 (PS3) days pod storage, without (D0) or with (D1) depulping, and after fermentation for 5 or 6 days (d), from experimental runs A and B.

In order to explain the differences seen in the sensory analysis between the five cocoa liquors from experimental runs A and B, a multivariate analysis was conducted. First, looking at all samples together and at the graphical representation from the principal component analysis and cluster analysis of the sensory data (Figure 11), the liquors could be divided into three groups. Sample B_PS1D0d6 represents the first cluster, with its intense acidity and fruity-citrus aroma notes. Cluster 2 is represented by samples A_PS1D0d5 and A_PS1D1d5, with their intense cocoa and nutty aroma notes. Finally, cluster 3 is represented by the two samples B_PS1D1d6 and B_PS3D1d6, showing intense bitterness and medium-high astringency.

**Figure 12:**
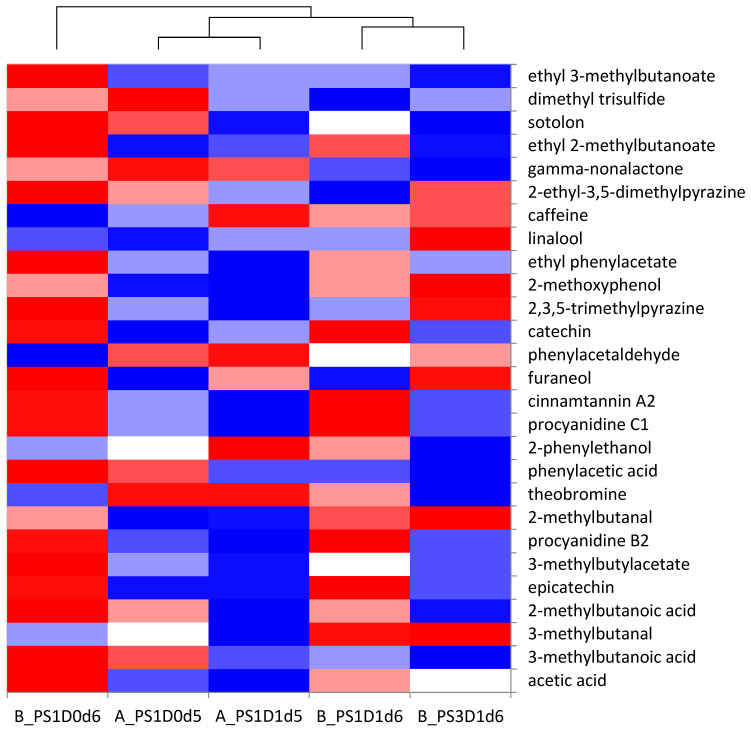
Heat map of the key aroma and tastant compound concentrations found in the cocoa liquors from the experimental runs A and B, with 1 (PS1) or 3 (PS3) days pod storage, without (D0) or with (D1) depulping, and fermented for 5 or 6 days (d). Color scale from blue to red through white (blue: lowest concentration; red: highest concentration).

The chemical analyses of selected key aroma and tastants were combined in a heat map (Figure 12). It can be observed that the same clustering of the samples occurs as for the sensory analysis. The cocoa liquor B_PS1D0d6, forming the first cluster, was characterized by higher amounts of acetic acid, ethyl phenylacetate, ethyl 2-methylbutanoate and ethyl 3-methylbutanoate, explaining the acidity and fruitiness. The liquors from cluster 2 (A_PS1D1d5 and A_PS1D0d5) differentiated themselves from the others as to the attributes cocoa and nutty. Based on the chemical analyses, those two samples showed less acetic acid, less 2- and 3-methylbutanoic acid (pungent), less ethyl 2 and 3-methylbutanoate (fruity) and less ethyl phenylacetate (flowery), compared to the other liquors. One explanation would be that the lower amounts of pungent and fruity/flowery notes increased the perception of the cocoa and nutty notes of the liquors A_PS1D1d5 and A_PS1D0d5. The third cluster (B_PS1D1d6 and B_PS3D1d6) was characterized in the sensory analysis by higher bitterness and astringency. This characteristic is well explained by the higher content of flavanols in the liquor B_PS1D1d6, but not by the lower flavanol content of the liquor B_PS3D1d6. 

## 4. Discussion

### 4.1. Influences of Depulping on Fermentation Dynamics and the Quality of the Dried Beans and the Cocoa Liquor

The depulping showed only minor influences during the fermentation process and on the dried cocoa beans. Most differences were observed at the beginning of the fermentation. A greater increase in temperature was observed within the first 58–63 h of fermentation in the depulped beans, compared to the non-depulped beans, although the difference was not significant. This trend of a slightly quicker temperature increase at the beginning of fermentation was also observed in other studies investigating pulp-preconditioning methods, such as pod storage [9]. 

With lower pulp content at the beginning of the fermentation, the anaerobic phase of fermentation may have been suppressed [11], and this could have led to a slightly accelerated fermentation. However, only yeasts and lactic acid bacteria were enumerated (Supplementary Material, Tables S2 and S3), and this assumption cannot be clearly confirmed by the lower observed cell counts of the lactic acid bacteria in depulped samples. This aerobic environment is favorable for the growth of acetic acid bacteria, which produce acetic acid from ethanol. Since this reaction is exothermic [25], it could explain the slight increase of the temperature observed in the depulped beans at the beginning of the fermentation [25]. Nevertheless, a negative correlation between the pH of cotyledon and temperature can be observed on day 2 of fermentation (*r* = −0.56, *p* < 0.05) for all samples, while depulped beans (with 1-day pod storage) showed lower pH and higher temperatures. This can be explained, with the assumption of accelerated fermentation, by the production of acetic acid by acetic acid bacteria starting earlier in depupled beans and diffusing into the cotyledon [25], which would result in the reduction of pH. The same correlation was found in Streule et al. [9] and, also, lower pH values were measured by Bariah et al. [10], where pod storage was examined, assuming that it has a similar effect to depulping. In the further course of the fermentation, no significant differences or trends could be observed in samples without versus with depulping. This aligns with the results as to viability of the embryo at the end of fermentation, but also with the cut-test of the dried beans, where no significant differences were detected between depulped and non-depulped beans. 

Interestingly, lower amounts of organic acids could be found at the end of fermentation (e.g., less lactic acid in A_PS1D1d5 than A_PS1D0d5 and less acetic acid in C_PS1D1d7 than C_PS1D0d7) in some depulped samples. The drying process allows volatile acids, such as acetic acid, to evaporate, while non-volatile acids, such as lactic acid, remain in the bean [13,20]. This was confirmed by the lower acetic acid content in the dried beans compared to the content measured in the beans at the end of fermentation. Some samples in experimental runs A and B had lower levels of lactic acid, but the reason for this remains unclear. In depulped samples, there were lower amounts of lactic acid and acetic acid compared to non-depulped samples (e.g., A_PS1D1d5 vs. A_PS1D0d5; B with depulping vs. without depulping, regardless of pod storage time; and in C, significantly less acetic acid in depulped samples, regardless of fermentation time). Additionally, a trend of slightly higher pH values was noted in depulped dried samples compared to non-depulped samples. This supports the findings from previous studies that a pulp pre-conditioning step could reduce acidity in dried beans [7,8,9,10,11,12,25,32]. 

No significant differences were observed in the cut-test regarding depulping. Differences in the fermentation index between depulped and non-depulped samples were only observed in experimental run B, though the reason for this is unclear. For example, fewer violet beans were found in depulped samples with a 1-day pod storage time compared to non-depulped samples. These findings indicate that depulping had only minor effects on fermentation dynamics and the quality of dried beans. 

Significant differences were observed when comparing similar samples from 2022 and 2023 (specifically, PSD1d5 and PS1D0d5 in experimental runs A and C). Differences were noted in cotyledon pH and sucrose levels at the beginning of fermentation, as well as the ambient temperature during the day during fermentation. These discrepancies may be attributed to variations in genetic material or different conditions during fruit maturation. It is crucial to consider the influences of these factors and their potential impacts on the cocoa bean’s flavor. Additionally, differences in the fermentation index, which relates to the brown color inside the bean, were observed. The higher fermentation index in the 2023 beans suggests different internal processes having occurred during fermentation. Measuring polyphenols in dried beans would have been advantageous in better understanding these variations. Despite these differences, the trends and influences related to depulping parameters remained consistent across both years.

In contrast, the analysis of the cocoa liquors revealed some differences between samples with and without depulping. The analysis of the key odorants showed differences in specific compounds between the cocoa liquors made from depulped beans and the ones made from non-depulped beans. With regards to key tastant composition, there were no differences. Overall, no negative effect of depulping on the flavor profile was found during the sensory evaluation of the liquors. The major impact of depulping was observed in the intensity of acidity and fruity-citrus notes, and confirms the observation made by Haruna et al. [33]. The cocoa liquors made from the depulped beans showed less fruity-citrus notes and acidity than the cocoa liquors made from the non-depulped beans, aligning with the results observed in the dried beans, with rather lower levels of acidity in depulped compared to the non-depulped samples. These differences can be explained on the chemical level by the lower concentrations of volatile acids (acetic acid (pungent), 2- and 3-methylbutanoic acids (pungent)), esters (ethyl phenylacetate (flowery) and 3-methylbutylacetate (fruity)) and pyrazines (2,3,5-trimethylpyrazine (earthy, roasty), 2-ethyl-3,5-dimethylpyrazine (earthy)) in the liquors produced from the depulped beans. 

The cocoa pulp plays an important role during fermentation. First bioconverted by yeast, then by lactic and acetic acid bacteria, important compounds such as acetic acid and lactic acid are formed from the pulp components [27]. Permeating the cocoa seed tissue, this creates a rise in temperature in the fermentation mass, leading to an activation of enzymes that degrade proteins and carbohydrates, leading to the release of aroma and flavor precursors such as reducing sugars and free amino acids [27,28,47]. Moreover, the pulp itself is rich in aromatic compounds [28]. As the migration of aromatic compounds from the pulp to the beans is assumed to play an important role, notably as a reservoir for fine flavor cocoa [28,48], an impact of depulping on the overall cocoa aroma composition was expected. The differences observed in this study between the depulped and non-depulped samples on the chemical level could be explained by different factors. Among key odorants quantified, esters are a class of compounds playing an important role in the aroma of cocoa products. Already present in low quantities in the unfermented cocoa beans [49] and in the pulp of freshly opened cacao pods [28], most esters found in cocoa are higher after fermentation (due to yeast activity) or after incubation of the cocoa beans [49,50,51,52,53,54,55]. The cocoa pulp serves as basis for the development of microbial strains with pectinolytic activities that synthesize important aroma and flavor precursors, as well as aroma compounds, during fermentation, among them, esters [50,51,54]. As no difference was observed in the yeast cell count during fermentation between depulped and non-depulped beans (Supplementary Material, Table S2), the lower ester content found in the liquors made from depulped beans could therefore be explained by a lower rate of ester infusion from the pulp to the beans during the fermentation.

Some differences were also seen in the pyrazine contents of the depulped and non-depulped samples. The majority of pyrazines result from the Maillard reaction [49,56]. Alpha-aminoketones, precursors of pyrazines, are formed from reducing sugars and free amino acids during the Maillard reaction that occurs mainly during the roasting and, to a lesser extent, during the drying of the beans. As the roasting and drying parameters were constant in this study, the differences in the pyrazine content observed could be due only to a difference in terms of precursors formed in the depulped and non-depulped cocoa beans. A lower amount of reducing sugars and free amino acids in the depulped and fermented beans could explain the lower content of pyrazines found in the liquors, as less alpha-aminoketones would have been formed by the Maillard reaction. The content of reducing sugars formed during fermentation was not significantly different. However, the free amino acid content of the depulped and non-depulped beans was not investigated during this study. Therefore, only an assumption can be made, specifically, that the fermentation of depulped beans leads to the release of fewer free amino acids, in comparison to the non-depulped beans. To our knowledge, there is currently no literature describing the free amino acid content in depulped cocoa beans, and this should therefore be examined in a future study.

Pyrazines are also formed from di- and tripeptides during the Maillard reaction [57]. The lower concentrations of pyrazines found in the depulped liquors could be postulated from the lower amounts of organic acids found at the end of the fermentation in the depulped samples. With less acidity reached in the cotyledon during the fermentation, and given the higher pH found in the depulped dried beans, it could be assumed that the endopeptidases might not have been fully activated, resulting in lower amounts of the precursors of pyrazines, such as small di- and tripeptides, in the depulped beans [58].

Lastly, the differences in volatile acid concentrations between the depulped and non-depulped samples can be explained by the lower pulp volume available for fermentation. As described by Meyer et al. [11], a reduced pulp-volume leads to an enhanced mass aeration during fermentation, increasing the ratio of respiration to ethanol fermentation and consequently lowering the amount of acetic acid produced by the microorganisms.

### 4.2. Influences of Pod Storage on Fermentation Dynamics and the Quality of Dried Beans and Cocoa Liquor

The impact of pod storage on the fermentation process and the quality of dried beans was minor, showing no significant differences in the cut-test and fermentation index. Similar to the influence observed in depulping, pod storage also displayed tendencies, especially regarding temperature and pH development, during fermentation. Samples stored for 3 days showed a tendency for higher temperatures from day 2 to day 4 of fermentation, indicating a slightly accelerated process, possibly due to lower pulp content, which was not measured in this study but has been observed in previous studies investigating the influence of pod storage [9,10].

The assumption of lower pulp content suggests that the anaerobic phase of fermentation might be suppressed, creating a better environment for acetic acid bacteria to oxidize ethanol to acetic acid, as already described in Section 4.1 [9]. Despite this, no differences were detected in acetic acid levels at the end of fermentation or in the dried beans. However, significantly lower lactic acid levels were found in dried beans with 3-day pod storage compared to 1-day, likely due to a shorter anaerobic phase and a reduced activity of lactic acid bacteria, which cannot be confirmed with cell count data (Supplementary Material, Table S3). Interestingly, lactic acid was slightly increased in the 3-day sample compared to the 1-day sample at the end of fermentation, though no explanation for this observation was found.

Additionally, samples with 3-day pod storage tended to have lower pH levels on fermentation days 2 and 3 compared to the 1-day storage, further indicating an accelerated fermentation. Despite these tendencies, no further differences were detected in quality indicators such as sugars or the fermentation index, which is consistent with findings by Streule et al. [9]. Additionally, no differences were observed in the cut-test, which contrasts with previous studies that reported improved results with pod storage [7,8,9].

Even though only minor differences were detected during fermentation and only one cocoa liquor was produced from the beans of the pods stored for 3 days, the results from the sensory analysis as well as the chemical analysis indicate that the pod storage time might be a critical parameter. Indeed, the sensory analysis revealed the presence of earthy/mushroom-like off-flavors in the cocoa liquors made of the beans with 3-day pod storage. This off-flavor was not found in the liquors of the beans from the 1-day pod storage. Moreover, the longer pod-storage showed to have an influence on the bitterness and astringency, as well as on the acidity of the cocoa liquor. On the chemical level, a longer pod storage time (3 days vs 1 day) led to an increase in the pyrazine concentration and a decrease in all flavanols (DP 1–4) analyzed, as well as lower contents of volatile acids (acetic acid, 2- and 3-methylbutanoic acid and phenylacetic acid) and esters.

The influence of pod storage on the beans’ chemical composition has already been studied. Afoakwa et al. [59] reported that a longer pod storage time decreases the total phenolic content of the cocoa beans. After harvest, the pods remain as living systems in which metabolic processes and biochemical changes still occur [5,60]. The assumption was made that the decrease in polyphenol content occurs through the activation of the polyphenol oxidase enzyme during a longer pod storage [59]. 

The impact of pod storage on the aroma composition of cocoa liquors and chocolates was also previously studied [5,18,61]; in these studies, the influence of roasting temperatures was also taken into account in relation to the formation of volatile compounds. Referring to the data generated with milder roasting temperatures, the results presented here are in line with some of the findings by Hinneh et al. [5,61], which demonstrated that a longer pod storage leads to a lower concentration of acetic acid and higher concentrations of pyrazines, whereas a significant increase in 2-methoxyphenol but not of dimethyltrisulfide was also found in our samples. According to Meyer et al. [11], the lower concentration of acetic acid found in the 3-day pod storage liquor could be explained by a reduced pulp volume, which would enhance aeration during fermentation and increase respiration relative to ethanol fermentation, as well as promote acetic acid oxidation, thus reducing seed acidification. However, the concentration of acetic acid in the dried beans was not significantly different between 1- and 3-day pod storage.

The lower concentration of esters cannot be clearly explained by the yeast cell counts (Supplementary Material, Table S2). Esters are present at low quantities in the unfermented cocoa beans [49] and in the pulp of freshly opened cacao pods [28]. Hamdouche et al. [18] concluded that shorter pod storage times would promote the presence of esters, as longer pod storage would lead to a substrate reduction that would impair the synthesis of esters. 

### 4.3. Influence of Fermentation Time on the Quality of Dried Beans and Cocoa Liquor

During the post-harvesting period, microbial activities occur in the pulp, and biochemical transformations occur in the cotyledon [54,62]; these are processes that require sufficient time. Therefore, the fermentation time is a crucial parameter to be considered during the post-harvesting processing, specifically, as to how it impacts the final quality (e.g., cut-test and aroma of cocoa and cocoa products) [18,49,63,64,65]. It is known that pulp pre-conditioning methods might lead to accelerated fermentation [9,10], as indicated by a faster temperature increase. Therefore, fermentation time was investigated as to the possibility of shortening its duration, in the period after depulping is performed. This aligns with the suggestion of Schwan and Wheals [25] that mechanical removal of pulp could enhance fermentation within a shorter timeframe.

Incomplete or weak fermentation (e.g., caused by unsuitable fermentation devices or methods) can lead to insufficient degradation of sucrose in the cotyledon and can result in elevated sensory attributes, such as bitterness or astringency [17]. Furthermore, the pH of the cotyledon in dried beans remains high, indicating inadequate acidification, a process which is essential for the development of important flavor precursors [17,47,66,67].

As described in previous studies, the maximal temperature during fermentation should be ≥40 °C in order to obtain an acceptable final bean quality [17], but typically reaches 45–50 °C [62,68,69]. In this study, average maximum temperatures exceeding 40 °C were reached by day 2 (run C) or day 3 (runs A, B). However, one exception was observed: in variation A_PS1D1d3, slightly lower maximum temperatures occurred (39.4 ± 5.4 °C). There is no clear explanation for this anomaly. In this study, the maximum temperature attained (Tmax) alone cannot indicate whether fermentation was insufficient and therefore part of a process leading to less well-fermented beans and a higher proportion of slaty beans. Similar maximum temperatures were observed, regardless of the fermentation time, yet there were fewer well-fermented beans and a higher proportion of slaty beans in the shorter fermented samples, such as A_PS1D1d3, compared to other variations from run A, or the 5-day versus 7-day samples from run C. This contrasts with the findings of Streule et al. [41], in which higher Tmax values were associated with a higher number of well-fermented beans. Therefore, it is suggested that the duration of maintaining the temperature might be crucial in this study. 

Other results also indicated that fermentation time is crucial, especially if too short. The pH of the cotyledon at the end of fermentation showed significant differences, particularly when fermented for only 3 or 4 days. Additionally, the higher pH value suggests that the process had not advanced enough for acids to diffuse inside the beans and inactivate the embryo [25]. This can be further supported by the observation that slightly less lactic and acetic acids diffused into the cotyledon, compared to longer fermentation periods in experimental run A. Consequently, these samples showed only 19 ± 23% inactivated embryos, correlating with the cotyledon pH (*r* = −0.85, *p* < 0.05) and, to a lower extent, with lactic acid levels (*r* = −0.63, *p* < 0.05) at the end of fermentation. Regarding the comparison of 5 days versus 7 days in experimental run C, no clear differences were observed in pH at the end of fermentation, embryo viability, or organic acid content. Furthermore, the sucrose level in the cotyledon of samples fermented for 3 or 4 days was significantly higher at the end of fermentation and in dried beans compared to those fermented for 5 days. This is a further indicator of incomplete fermentation and could lead to bitter and astringent flavors, as mentioned by Streule et al. [17]. These samples were also described as strongly bitter and astringent during sensory screening. However, the samples fermented for shorter times were not sensorially characterized in this study. In the dried beans, 3 and 4 days of fermentation (experimental run A) were insufficient to achieve determinations of less than 3% slaty beans by count, thus failing to meet the Grade 1 standards set by the Quality Control Division of the Ghana Cocoa Board [14]. Further studies in Ghana [16,70] observed similar results, indicating that fermentation duration is a crucial factor for cut-test outcomes. Amoah et al. [16] also observed that fermentation durations of 3 and 4 days resulted in substandard grades of cocoa.

In experimental run C, significant differences in the cut-test between 5- and 7-day fermented samples were observed. Fermentation for 5 days resulted in significantly jigher numbers of slaty and violet beans and significantly fewer well-fermented beans compared to 7 days. Depulping had no effect on these outcomes. Interestingly, in all variations, the cut-test revealed a composition of more than 3% slaty beans, regardless of depulping. The reason for this remains unclear and warrants further investigation. The results from the cut-test were consistent with those from the fermentation index. The fermentation index increased with longer fermentation times (3 or 4 days vs. 5 days in run A, and 5 vs. 7 days in run C). This significant impact of fermentation time on the fermentation index was also observed by Gunam et al. [71], who measured the intensity of the brown color in cocoa beans caused by the polyphenol oxidation during fermentation [71,72].

As already described in previous studies, the fermentation time is one of the parameters along the transformation steps of cocoa showing the biggest impact on the aroma of cocoa and cocoa products [18,49,63,65]. During fermentation, oxidation and polymerization reactions occur [29]. With longer fermentation times, lower amounts of the low molecular-weight polymers are found in the cocoa beans, as higher molecular-weight polymers are formed, thus reducing the bitterness and astringency of the cocoa [63]. Comparing the fermentation times in experimental run C, the liquors made with the beans fermented for 7 days showed significantly lower concentrations of monomers, dimers and trimers, compared to the liquors made from the beans fermented for 5 days. This finding correlated with a higher polymerization degree noted in beans fermented for longer times. Regarding the key aroma compounds quantified in this study, a fermentation of 7 days led to a lower concentration in volatile acids; an increase in Strecker aldehydes; and a lower total concentration of key aroma compounds. An increase of the aldehyde concentration with increased fermentation times has previously been reported [18,49,65]. 2-Methylbutanal and 3-methylbutanal are known to be released from the amino acids isoleucine and leucine during thermal treatment through the Strecker degradation, and are responsible for the malty, chocolate-like aroma of cocoa [73]. Both key compounds increased in concentration after 7 days of fermentation, in both liquors made from depulped beans and those made from non-depulped beans. As lactic acid bacteria play a role in the release of free amino acids during fermentation, a longer fermentation time would increase the release of those important precursors, thereby promoting the formation of 2- and 3-methylbutanal. 

Rodriguez-Campos et al. [49] reported an increase of acetic acid during the first 4 days of fermentation, observing a decrease after 6 days. The fermentation conducted in the experimental run C seemed to follow the same trend, as a lower acetic acid content was found in the liquors from beans fermented for 7 days compared to those fermented for 5 days. 

The lower total concentration of key aroma compounds in the 7-day fermentation samples, together with increased concentrations of 2-methoxyphenol (smoky) and dimethyl trisulfide (cabbage-like) could indicate that a 5-day fermentation would be sufficient to produce good quality cocoa, with or without a depulping step before fermentation. 

Nevertheless, it is important to combine all results (e.g., cut-test, aroma and sensory) and consider the quality (cut-test) standards set by the Quality Control Division of the Ghana Cocoa Board [14]. For instance, a shorter fermentation time results in e.g., fewer well-fermented beans. However, sensory characteristics must be evaluated before drawing final conclusions about the 7-day fermentation period. Additionally, it is recommended to conduct simultaneous fermentations, including one with a 6-day period, as independent repetitions have shown significant influences on the results, as previously observed by Streule et al. [9]. This is evident in some outcomes (e.g., viability of embryos), due to the large standard deviations observed.

## 5. Conclusions

This study investigated the potential for valorizing cacao fruit by mechanically removing 17–20% of cocoa pulp, relative to the total bean-pulp-mass weight. The results indicate that this partial depulping can enhance farmers’ income without compromising cocoa bean quality, provided that the correct pod storage time (1 day) and fermentation time (5–6 days) are chosen.

Key findings include no adverse effects of depulping on cocoa bean quality, with regards to dried beans and cocoa liquor, with 1-day pod storage and 5–6 days fermentation. Extending fermentation to 7 days did not negatively impact the cocoa bean quality with regards to the organic acids present. However, decreases in the concentrations of key tastants and key aroma compounds were found in both depulped and non-depulped samples. Therefore, the impact on the overall sensory profiles would need to be evaluated before recommending the 7-day fermentation.

Three-day pod storage, combined with depulping and 6 days of fermentation, can lead to undesirable earthy or mushroom-like off-flavors. Despite this, beans with 3-day pod storage showed a slightly higher temperature in the cocoa-pulp mass, suggesting a more thorough fermentation process. Further research is required to explore methods to reduce fermentation time while maintaining a 3-day pod storage period and depulping.

This study highlights the importance of a minimum 5-day fermentation period, with or without depulping, to meeting Ghanaian quality standards, particularly regarding the cut-test. A shorter fermentation duration of 3 or 4 days significantly increased the occurrence of violet and slaty beans and is therefore not recommended.

Furthermore, we highlight the influences of independent runs and harvest periods on the experimental results, likely due to varying climatic conditions during cacao growth and post-harvest processes. For instance, in the 2022 experimental runs, ambient daytime temperatures were slightly lower than in 2023. However, the trends and influences of parameters like depulping and fermentation time remained consistent across both years. Monitoring humidity and precipitation in future studies could provide additional insights into the climatic factors affecting cacao quality.

In conclusion, this study opens new avenues for enhancing cocoa bean processing through partial depulping, with specific recommendations for pod storage and fermentation times to ensure quality and maximize farmer income. Additionally, the study suggests that it may be possible to remove more pulp without comprising quality, but further research is needed to confirm this assumption.

## Figures and Tables

**Table 1:** Overview of investigated post-harvest process variations.

Experimental Run	Fermentation Variation	Total Repetitions ^1^	Pod Storage (Days)	Depulping Process	Fermentation Time (Days)	Fermentation Time (h)
A	PS1D1d3	*n* = 2 × 2	1	with	3	64 (run 1); 69 (run 2)
	PS1D1d4	*n* = 2 × 2	1	with	4	92 (run 1); 91 (run 2)
	PS1D1d5	*n* = 2 × 2	1	with	5	116 (run 1); 115 (run 2)
	PS1D0d5	*n* = 2 × 2	1	without	5	
B	PS1D0d6	*n* = 2 × 2	1	without	6–6.5	156 (run 1); 147 (run 2)
	PS1D1d6	*n* = 2 × 2	1	with	6–6.5	
	PS3D0d6	*n* = 2 × 2	3	without	6–6.5	
	PS3D1d6	*n* = 2 × 2	3	with	6–6.5	
C	PS1D0d5	*n* = 2 × 3	1	without	5	120 (run 1), 116 (run 2), 118 (run 3)
	PS1D1d5	*n* = 2 × 3	1	with	5	
	PS1D0d7	*n* = 2 × 3	1	without	7	169 (run 1), 169 (run 2), 165 (run 3)
	PS1D1d7	*n* = 2 × 3	1	with	7	

^1^ Time-dependent repetitions × time-independent runs.

**Table 2:** Sensory vocabulary, definition, and scale anchor points.

Category	Attributes	Definition	Scale
Basic taste	Acidity	Basic taste, perception of the intensity of acidity like a solution of citric acid or acetic acid	Low-intense
	Bitterness	Basic taste, perception of the intensity of bitterness like a caffeine solution	Low-intense
Trigeminal sensation	Astringency	Dry and rough mouthfeel	Low-intense
Aroma (retronasal)	Fruity-citrus	Aroma perception, reminiscent of citrus fruits and bergamot	Low-intense
	Fruity-dried fruits	Aroma perception, reminiscent of dried fruits, like raisins	Low-intense
	Cocoa	Aroma perception, reminiscent of cocoa powder	Low-intense
	Nutty	Aroma perception, reminiscent of ripe, unroasted nuts	Low-intense
	Woody	Aroma perception, reminiscent of dry wood	Low-intense
	Spicy	Aroma perception, reminiscent of vanilla or tobacco	Low-intense
	Herbal	Aroma perception, reminiscent of dried herbs, like vermouth	Low-intense
Basic taste	Roasty	Aroma perception, reminiscent of roasted bread / toasted bread	Low-intense

**Table 3:** Information on calibration of selected polyphenols and alkaloids.

Compound	Detection/Quantification	Linear Range (µg/mL)	Calibration Equation	R2
Catechin	MS/EIC	1–100	y = 309,070x + 3 × 10^6^	0.9923
Epicatechin	MS/EIC	1–100	y = 293,036x + 3 × 10^6^	0.9985
Procyanidine B2	MS/EIC	1–25	y = 247,405x + 212,827	0.9978
Procyanidine C1	MS/EIC	1–25	y = 93,519x + 16,054	0.9974
Cinnamtannin A2	MS/EIC	1–100	y = 21,900x + 15,453	0.9968
Theobromine	UV/275 nm	1–600	y = 9.2645x + 56.775	0.9978
Caffeine	UV/275 nm	1–300	y = 8.4225x − 0.5098	1.0000

**Table 4:** Development of cotyledon pH during fermentation days (d0, d1, d2, d3, d4, d5, d6 and d7) and of dried beans (dried) samples with 1 (PS1) or 3 (PS3) days pod storage, without (D0) or with (D1) depulping, and fermented for 3, 4, 5, 6 or 7 days (d), from experimental runs A (*n* = 4), B (*n* = 4; * *n* = 2) and C (*n* = 3).

Variation		d0	d1	d2	d3	d4	d5	d6	d7 ^1^	Dried
Run A	PS1D1d3	6.57 ± 0.03 ^a^	6.44 ± 0.03 ^a^	6.33 ± 0.05 ^b^	6.10 ± 0.31 ^a^	-	-	-	-	5.87 ± 0.45 ^a^
	PS1D1d4	6.57 ± 0.03 ^a^	6.47 ± 0.02 ^a^	6.39 ± 0.03 ^b^	6.09 ± 0.22 ^a^	5.67 ± 0.55 ^a^	-	-	-	5.79 ± 0.16 ^a^
	PS1D1d5	6.58 ± 0.02 ^a^	6.47 ± 0.05 ^a^	6.35 ± 0.04 ^b^	6.06 ± 0.20 ^a^	5.63 ± 0.50 ^a^	5.02 ± 0.32 ^a^	-	-	5.72 ± 0.32 ^a^
	PS1D0d5	6.43 ± 0.19 ^a^	6.48 ± 0.03 ^a^	6.48 ± 0.04 ^a^	5.72 ± 0.47 ^a^	5.15 ± 0.29 ^a^	4.78 ± 0.26 ^a^	-	-	5.42 ± 0.24 ^a^
Run B	PS1D0d6	6.51 ± 0.02 ^b^	6.47 ± 0.02 ^a^	6.46 ± 0.03 ^a^	6.07 ± 0.21 ^a^	5.21 ± 0.24 ^a^	4.62 ± 0.02 ^a^	4.56 ± 0.04 ^a^	4.62 ± 0.00 *	5.12 ± 0.08 ^b^
	PS1D1d6	6.53 ± 0.04 ^ab^	6.40 ± 0.03 ^ab^	6.21 ± 0.11 ^ab^	5.61 ± 0.71 ^a^	5.15 ± 0.62 ^a^	4.64 ± 0.13 ^a^	4.63 ± 0.11 ^a^	4.70 ± 0.15 *	5.24 ± 0.10 ^ab^
	PS3D0d6	6.54 ± 0.03 ^ab^	6.36 ± 0.07 ^ab^	6.23 ± 0.15 ^ab^	5.10 ± 0.29 ^a^	4.75 ± 0.22 ^a^	4.55 ± 0.07 ^a^	4.54 ± 0.05 ^a^	4.63 ± 0.22 *	5.23 ± 0.06 ^ab^
	PS3D1d6	6.59 ± 0.02 ^a^	6.33 ± 0.10 ^b^	5.75 ± 0.58 ^b^	5.16 ± 0.63 ^a^	4.87 ± 0.39 ^a^	4.68 ± 0.10 ^a^	4.73 ± 0.19 ^a^	4.69 ± 0.03 *	5.45 ± 0.16 ^a^
Run C	PS1D0d5	6.44 ± 0.02 ^a^	6.36 ± 0.09 ^a^	6.08 ± 0.03 ^a^	4.84 ± 0.10 ^a^	4.75 ± 0.07 ^a^	4.83 ± 0.16 ^a^	-	-	5.23 ± 0.08 ^a^
	PS1D1d5	6.46 ± 0.06 ^a^	6.27 ± 0.07 ^a^	5.73 ± 0.31 ^a^	4.80 ± 0.13 ^a^	4.78 ± 0.13 ^a^	4.96 ± 0.16 ^a^	-	-	5.45 ± 0.15 ^a^
	PS1D0d7	6.38 ± 0.03 ^a^	6.30 ± 0.08 ^a^	6.02 ± 0.09 ^a^	4.83 ± 0.07 ^a^	4.71 ± 0.20 ^a^	4.88 ± 0.30 ^a^	4.89 ± 0.16 ^a^	5.14 ± 0.49 ^a^	5.54 ± 0.23 ^a^
	PS1D1d7	6.44 ± 0.07 ^a^	6.27 ± 0.06 ^a^	5.71 ± 0.36 ^a^	4.92 ± 0.19 ^a^	4.86 ± 0.04 ^a^	4.93 ± 0.22 ^a^	4.93 ± 0.07 ^a^	5.33 ± 0.23 ^a^	5.65 ± 0.16 ^a^

Values in same column for the same run with same superscript letters are statistically equal. Values represent the mean ± standard deviation. ^1^ In experimental run B, run 1 started at 7.30 pm at d0 and was fermented until d7 (8.15 am); run 2 started earlier (3 pm), and therefore, the last analysis was performed on d6 (8 am) and the drying started in late afternoon (4 pm), resulting in * *n* = 2 at d7 (no ANOVA was performed).

**Table 5:** Maximum temperature (°C) per fermentation day (d0, d1, d2, d3, d4, d5, d6 and d7) of samples with 1 (PS1) or 3 (PS3) days pod storage, without (D0) or with (D1) depulping, and fermented for 3, 4, 5, 6 or 7 days (d) and average ambient temperature during the day (6am-6pm) and night (6pm-6am) from experimental run A (*n* = 8), B (*n* = 8), C (*n* = 12).

Variation		d0	d1	d2	d3	d4	d5	d6	d7 ^1^
Run A	PS1D1d3	29.2 ± 1.5 ^a^	33.2 ± 1.6 ^ab^	37.6 ± 4.1 ^a^	39.4 ± 5.4 ^b^	-	-	-	-
	PS1D1d4	30.1 ± 2.1 ^a^	34.3 ± 1.4 ^a^	39.6 ± 3.4 ^a^	44.4 ± 2.2 ^a^	44.0 ± 0.8 ^a^	-	-	-
	PS1D1d5	29.4 ± 1.8 ^a^	33.3 ± 1.5 ^ab^	37.7 ± 3.5 ^a^	42.4 ± 3.0 ^ab^	43.7 ± 2.5 ^a^	43.4 ± 2.5 ^a^	-	-
	PS1D0d5	28.1 ± 0.5 ^a^	32.3 ± 0.8 ^b^	36.5 ± 3.1 ^a^	43.4 ± 2.4 ^ab^	45.6 ± 1.3 ^a^	44.6 ± 1.5 ^a^	-	-
	Ambient	26.7 ± 2.8 (6 a.m.–6 p.m.) and 22.9 ± 0.7 (6 p.m.–6 a.m.)							
Run B	PS1D0d6	28.0 ± 2.7 ^a^	33.2 ± 1.5 ^b^	37.1 ± 1.7 ^b^	42.8 ± 2.3 ^a^	46.2 ± 1.8 ^ab^	47.2 ± 1.1 ^ab^	44.5 ± 0.6 ^a^	44.4 ± 0.3 ^a^
	PS1D1d6	29.3 ± 3.0 ^a^	35.0 ± 1.1 ^ab^	39.0 ± 2.7 ^ab^	43.9 ± 2.5 ^a^	45.0 ± 3.2 ^b^	46.3 ± 2.6 ^b^	43.9 ± 0.9 ^a^	43.0 ± 1.0 ^a^
	PS3D0d6	30.3 ± 3.3 ^a^	35.7 ± 1.5 ^a^	40.7 ± 2.8 ^ab^	45.5 ± 1.6 ^a^	48.4 ± 1.0 ^a^	48.6 ± 0.9 ^a^	44.8 ± 0.7 ^a^	43.9 ± 1.8 ^a^
	PS3D1d6	31.5 ± 1.3 ^a^	35.8 ± 1.7 ^a^	42.0 ± 3.1 ^a^	44.7 ± 3.0 ^a^	46.4 ± 1.4 ^ab^	47.0 ± 1.2 ^ab^	44.3 ± 1.3 ^a^	43.5 ± 2.5 ^a^
	Ambient	26.1 ± 4.1 (6 a.m.–6 p.m.) and 25.7 ± 4.1 (6 p.m.–6 a.m.)							
Run C	PS1D0d5	30.7 ± 1.9 ^a^	35.0 ± 2.5 ^c^	44.8 ± 2.8 ^a^	45.5 ± 1.6 ^a^	48.6 ± 1.3 ^a^	46.3 ± 2.0 ^a^	-	-
	PS1D1d5	32.2 ± 2.7 ^a^	38.0 ± 2.5 ^ab^	46.5 ± 2.2 ^a^	46.1 ± 1.5 ^a^	48.5 ± 1.2 ^a^	47.0 ± 1.6 ^a^	-	-
	PS1D0d7	31.0 ± 1.3 ^a^	35.3 ± 1.9 ^bc^	44.9 ± 2.4 ^a^	45.3 ± 1.6 ^a^	48.7 ± 0.9 ^a^	46.9 ± 1.2 ^a^	47.8 ± 2.1 ^a^	46.2 ± 2.6 ^a^
	PS1D1d7	32.5 ± 2.3 ^a^	38.3 ± 3.0 ^a^	46.3 ± 1.5 ^a^	45.7 ± 1.0 ^a^	48.0 ± 0.8 ^a^	47.2 ± 0.7 ^a^	46.9 ± 1.8 ^a^	46.0 ± 2.2 ^a^
	Ambient	29.6 ± 3.6 (6 a.m.–6 p.m.) and 24.4 ± 1.0 (6 p.m.–6 a.m.)							

Values in the same column for the same run with same superscript letters are statistically equal. Values represent the mean ± standard deviation. Maximum temperature is shown per day (d0: fermentation start until 6am; following days, 6 a.m. to 6 a.m.). ^1^ In experimental run B, run 1 started at 7.30 p.m. at d0 and was fermented until d7 (8.15 a.m.). Run 2 started earlier (3 p.m.); therefore, the last analysis was performed on d6 (8 a.m.), and the drying started in late afternoon (4 p.m.).

**Table 6:** Average concentrations of selected taste-active compounds in the cocoa liquors: alkaloids (theobromine and caffeine) and flavanols (catechin, epicatechin, procyanidine B2, procyanidine C1 and cinnamtannin A2) in experimental runs A, B and C, with 1 (PS1) or 3 (PS3) days pod storage, without (D0) or with (D1) depulping, and after fermentation for 5, 6 or 7 days (d), expressed as g or mg/kg fat-free dry matter (ffdm). Significant differences of REML analysis are shown in bold.

Sample Variations/Key Tastants	A_PS1D0d5	A_PS1D1d5	B_PS1D0d6	B_PS1D1d6	B_PS3D1d6	C_PS1D0d5	C_PS1D1d5	C_PS1D0d7	C_PS1D1d7	REML *p*-Values α = 5%	
	Selected alkaloids—Average concentration ± standard deviation (g/kg ffdm) *n* = 3									Depulping	Pod storage
Theobromine	29.93 ± 0.42	29.97 ± 0.12	29.02 ± 0.55	29.56 ± 0.44	28.49 ± 0.2	25.95 ± 0.19	27.56 ± 0.7	28.19 ± 1.24	27.49 ± 0.51	0.225	0.069
Caffeine	2.65 ± 0.05	2.71 ± 0.03	2.54 ± 0.03	2.68 ± 0.01	2.70 ± 0.01	3.21 ± 0.08	3.30 ± 0.13	3.11 ± 0.15	3.09 ± 0.08	0.051	0.318
	Selected polyphenols—Average concentration ± standard deviation (mg/kg ffdm) *n* = 3										
Catechin	0.43 ± 0.02	0.60 ± 0.06	0.76 ± 0.02	0.83 ± 0.01	0.54 ± 0.05	1.04 ± 0.01	1.03 ± 0.1	0.66 ± 0.02	0.66 ± 0.03	**0.024**	**<0.0001**
Epicatechin	2.06 ± 0.11	1.99 ± 0.2	3.14 ± 0.01	3.43 ± 0.13	2.18 ± 0.12	3.42 ± 0.05	3.05 ± 0.36	1.90 ± 0.03	1.80 ± 0.06	0.425	**<0.0001**
Procyanidine B2	1.65 ± 0.1	1.31 ± 0.15	2.67 ± 0.03	2.80 ± 0.08	1.71 ± 0.14	1.91 ± 0.04	1.89 ± 0.17	1.24 ± 0.02	1.17 ± 0.07	0.167	**<0.0001**
Procyanidine C1	1.50 ± 0.1	1.17 ± 0.15	2.09 ± 0.02	2.20 ± 0.09	1.38 ± 0.12	0.96 ± 0.03	1.04 ± 0.02	0.70 ± 0.03	0.74 ± 0.02	0.631	**<0.0001**
Cinnamtannin A2	1.50 ± 0.13	1.07 ± 0.19	2.08 ± 0.02	2.25 ± 0.09	1.38 ± 0.16	0.62 ± 0.05	0.76 ± 0.01	0.53 ± 0.04	0.60 ± 0.02	0.862	**<0.0001**
Total flavanols DP 1–4	7.14 ± 0.46	6.14 ± 0.75	10.75 ± 0.01	11.51 ± 0.41	7.20 ± 0.58	7.96 ± 0.09	7.77 ± 0.65	5.03 ± 0.04	4.98 ± 0.15	-	-

**Table 7:** Average concentrations of selected volatile compounds in experimental runs A, B and C, with 1 (PS1) or 3 (PS3) days pod storage, without (D0) or with (D1) depulping, and after fermentation for 5, 6 or 7 days (d), expressed as mg (^a^), µg (^b^) or ng/kg (^c^). Significant differences of REML analysis are shown in bold.

Sample	A_PS1D0d5	A_PS1D1d5	B_PS1D0d6	B_PS1D1d6	B_PS3D1d6	C_PS1D0d5	C_PS1D1d5	C_PS1D0d7	C_PS1D1d7	REML *p*-Values α = 5%	
Acids										Depulping	Pod storage
Acetic acid ^a^	1556.71 ± 69.30	1170.80 ± 92.85	2967.25 ± 98.17	2105.89 ± 117.88	1953.89 ± 130.89	3688.26 ± 55.23	3013.61 ± 103.78	2360.99 ± 44.18	2151.32 ± 38.19	**<0.0001**	**0.0077**
2-Methylbutanoic acid ^a^	12.70 ± 0.56	9.90 ± 0.29	14.39 ± 0.98	12.52 ± 0.16	10.11 ± 1.03	20.86 ± 0.23	18.82 ± 0.46	15.82 ± 0.35	14.30 ± 0.27	**<0.0001**	**<0.0001**
3-Methylbutanoic acid ^a^	35.05 ± 1.42	27.56 ± 0.76	39.28 ± 1.00	30.36 ± 0.79	25.11 ± 0.76	37.25 ± 0.22	33.97 ± 1.10	30.40 ± 0.46	29.69 ± 0.31	**<0.0001**	**<0.0001**
Phenylacetic acid ^a^	7.93 ± 0.57	7.18 ± 0.23	8.66 ± 0.17	7.17 ± 0.33	6.74 ± 0.26	79.79 ± 4.32	65.32 ± 3.87	52.44 ± 1.98	56.09 ± 3.20	0.0685	0.8835
Strecker—Aldehydes											
3-Methylbutanal ^a^	40.62 ± 4.26	37.44 ± 1.35	39.60 ± 1.88	42.20 ± 2.07	42.66 ± 4.07	50.77 ± 0.80	59.47 ± 5.77	79.49 ± 3.06	66.78 ± 6.04	0.7258	0.6935
2-Methylbutanal ^a^	3.85 ± 0.24	3.99 ± 0.18	4.65 ± 0.18	4.98 ± 0.21	5.30 ± 0.02	10.15 ± 0.05	11.69 ± 1.41	14.05 ± 0.70	14.04 ± 0.89	0.0661	0.6225
Phenylacetaldehyde ^a^	7.66 ± 0.72	7.89 ± 0.59	6.01 ± 0.35	7.16 ± 0.42	7.50 ± 0.47	4.91 ± 0.07	5.03 ± 0.19	5.06 ± 0.04	5.01 ± 0.10	**0.0458**	**0.0243**
Esters											
3-Methylbutyl acetate ^a^	2.33 ± 0.10	1.29 ± 0.07	5.88 ± 0.13	2.81 ± 0.07	1.73 ± 0.10	1.08 ± 0.05	0.79 ± 0.03	0.94 ± 0.04	0.70 ± 0.03	**<0.0001**	**0.0001**
Ethyl 2-methylbutanoate ^b^	32.19 ± 4.60	35.03 ± 3.33	55.63 ± 8.10	45.28 ± 2.30	32.20 ± 3.69	16.02 ± 0.42	20.57 ± 0.63	15.71 ± 0.27	16.00 ± 0.35	0.6999	**<0.0001**
Ethyl 3-methylbutanoate ^b^	46.64 ± 6.84	51.19 ± 6.17	77.97 ± 10.85	50.52 ± 3.70	43.82 ± 5.93	19.34 ± 1.00	20.04 ± 1.27	18.36 ± 1.15	16.10 ± 1.28	0.0670	**0.0052**
Ethyl phenylacetate ^b^	415.17 ± 25.66	291.50 ± 24.44	669.26 ± 35.12	498.49 ± 4.22	426.43 ± 25.86	342.32 ± 11.10	263.71 ± 5.27	242.64 ± 4.78	232.59 ± 2.62	**<0.0001**	**0.0003**
Pyrazines											
2,3,5-Trimethylpyrazine ^b^	424.39 ± 16.99	299.14 ± 12.32	574.89 ± 15.58	415.75 ± 19.09	561.17 ± 3.90	1376.35 ± 17.19	1141.55 ± 21.91	1156.36 ± 18.49	1108.41 ± 37.96	**<0.0001**	**0.0002**
2-Ethyl-3,5-dimethylpyrazine ^b^	353.73 ± 25.71	331.71 ± 22.22	387.70 ± 9.53	281.12 ± 15.72	373.53 ± 29.53	315.37 ± 12.85	270.22 ± 2.42	293.26 ± 11.03	282.43 ± 8.79	**<0.0001**	**0.0018**
Furans, furanones											
Furaneol ^a^	7.93 ± 0.28	8.45 ± 0.26	8.76 ± 0.25	8.03 ± 0.22	8.69 ± 0.35	55.18 ± 4.43	54.62 ± 3.41	55.52 ± 2.18	51.37 ± 0.61	0.8636	0.9439
Sotolone ^b^	27.27 ± 2.47	19.93 ± 1.08	29.97 ± 4.78	24.00 ± 1.92	19.11 ± 0.87	41.55 ± 2.89	36.97 ± 2.35	34.75 ± 1.10	33.10 ± 2.82	**<0.0001**	**0.0057**
2-Methyl-3-(methyldithio)furan ^c^	725.98 ± 63.19	553.00 ± 55.39	747.25 ± 42.97	501.72 ± 57.76	493.64 ± 66.29	2098.98 ± 52.84	2419.00 ± 186.34	2874.61 ± 272.22	2823.19 ± 333.31	0.6185	0.4108
Others											
2-Phenylethanol ^a^	14.12 ± 1.12	16.57 ± 1.30	13.55 ± 1.30	14.26 ± 0.52	10.90 ± 0.50	7.78 ± 0.31	8.03 ± 0.19	7.51 ± 0.42	7.99 ± 0.35	**0.0072**	**<0.0001**
2-Methoxyphenol ^b^	38.90 ± 0.42	16.35 ± 1.30	138.67 ± 2.27	140.27 ± 7.14	214.46 ± 16.59	209.59 ± 3.04	257.06 ± 6.33	390.27 ± 13.29	301.06 ± 1.53	0.1470	**0.0002**
Dimethyl trisulfide ^b^	48.76 ± 4.74	36.27 ± 6.49	41.49 ± 6.37	30.29 ± 3.20	37.24 ± 1.06	87.53 ± 1.18	102.51 ± 0.52	128.91 ± 3.92	142.72 ± 0.66	0.8264	0.8564
Linalool ^b^	873.51 ± 15.27	1017.69 ± 62.98	966.13 ± 68.22	1042.53 ± 17.71	1642.57 ± 117.73	1773.14 ± 43.08	1630.96 ± 16.15	1374.28 ± 28.87	1762.93 ± 28.85	**0.0124**	**<0.0001**
*Gamma*-nonalactone ^b^	118.81 ± 5.78	113.41 ± 16.81	106.41 ± 8.15	84.62 ± 11.81	67.55 ± 4.37	45.71 ± 5.03	47.35 ± 0.71	53.09 ± 4.43	56.77 ± 6.38	**0.0211**	**0.0005**
4-Methylphenol ^c^	409.42 ± 38.06	583.31 ± 22.38	332.60 ± 11.46	448.90 ± 30.35	403.12 ± 3.03	16.88 ± 0.64	16.03 ± 0.42	17.49 ± 0.23	17.09 ± 0.33	**0.0002**	0.4392

## Data Availability

The original contributions presented in the study are included in the article/Supplementary Material, further inquiries can be directed to the corresponding author.

## Supplementary Materials

The following supporting information can be downloaded at: https://www.mdpi.com/article/10.3390/foods13162590/s1, Figure S1: Fat content of the cocoa liquors, analyzed in triplicate; ns: no significant differences (ANOVA; *p* > 0.05); Table S1: Information on quantifier ions used and on calibration of selected key aroma compounds; Table S2: Cell counts of yeasts (log cfu/g) during fermentation days (d0, d2, d3, d4, d5, d6, d7) of samples with 1 (PS1) or 3 (PS3) days pod storage, without (D0) or with (D1) depulping and fermented for 3, 4, 5, 6 or 7 days (d) from experimental runs A (*n* = 2), B (*n* = 2; * *n* = 1) and C (*n* = 3,* *n* = 2); Table S3: Cell counts of lactic acid bacteria (log cfu/g) during fermentation days (d0, d2, d3, d4, d5, d6, d7) and of samples with 1 (PS1) or 3 (PS3) days pod storage, without (D0) or with (D1) depulping and fermented for 3, 4, 5, 6 or 7 days (d) from experimental runs A (*n* = 4), B (*n* = 4; * *n* = 2) and C (*n* = 3).
