# Supplementary material for: Influences of Depulping, Pod Storage and Fermentation Time on Fermentation Dynamics and Quality of Ghanaian Cocoa
Source: Foods. 2024 Aug 19;13(16):2590. doi: 10.3390/foods13162590 (PMC11354155; doi:10.3390/foods13162590)
Supplement: Supplementary file 1 [file foods-13-02590-s001.zip › foods-3127318-supplementary.pdf]

Supplementary Material

Measurement of Fat Content: Fat and water content of the cocoa liquors were determined in triplicate using a microwave and infrared moisture and solids analyzer coupled with a rapid NMR fat analyzer (Smart 6 + Oracle, CEM, Matthews, United States of America). Results are shown in Figure S1.

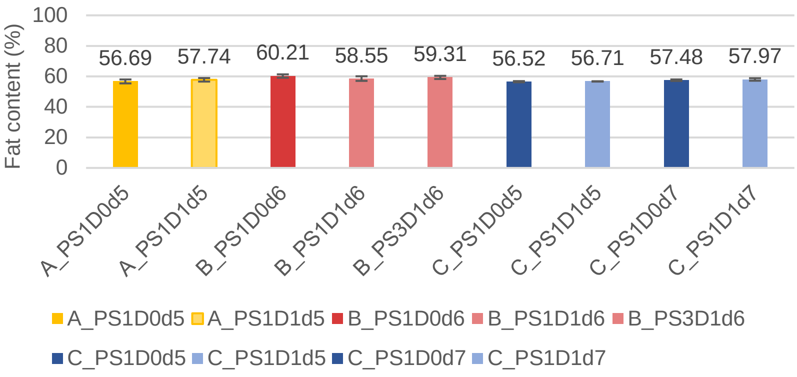

Figure S1. Fat content of the cocoa liquors, analyzed in triplicate; ns: no significant differences (ANOVA; $p > 0.05$).

Table S1. Information on quantifier ions used and on calibration of selected key aroma compounds.

**target compound**	**isotopically substituted internal standard**	**quantifier ions (m/z)**		**quantitation system**[a]	**calibration line**[b]	**R2**
		**analyte**	**standard**			
acetic acid	($^{13}C_2$)acetic acid	60	62	I	y = 0.519x + 0.0477	0.9987
2-methylbutanoic acid	3($^2H_3$)methyl-(2,2,3,4,4,4-$^2H_6$)butanoic acid	74	63	I	y = 1.1165x + -0.0026	0.9994
3-methylbutanoic acid	3($^2H_3$)methyl-(2,2,3,4,4,4-$^2H_6$)butanoic acid	60	63	I	y = 0.9073x + -0.0149	0.9999
phenylacetic acid	phenyl($^{13}C_2$)acetic acid	136	138	I	y = 0.9202x + 0.1431	0.9992
2-phenylethan-ol	2-($^2H_5$)phenylethanol	122	127	I	y = 1.0281x + 0.0067	0.9991
furaneol	4-hydroxy-2-methyl-5-(^{13}C)methyl(5-^{13}C)furan-3(2H)-one	128	130	I	y = 1.0392x + 0.0035	1.0000
3-methylbutanal	2-($^2H_3$)methylbutanal	71	73	II	y = 0.2171x + 0.1044	0.9964
2-methylbutanal	3-methyl(3,5-$^2H_2$)butanal	57	60	II	y = 0.8874x + 0.0126	0.9991
phenylacetaldehyde	phenyl($^{13}C_2$)acetaldehyde	120	122	II	y = 0.7446x + 0.0635	0.9997
3-methylbutylacetat	3-methylbutyl-($^{13}C_2$)acetate	61	63	II	y = 0.9541x + 0.0837	0.9999
2,3,5-trimethylpyrazin	2-($^2H_3$)methyl-3,5-dimethylpyrazine	122	125	I	y = 0.5082x + -0.0645	0.9981
2-ethyl-3,5-dimethylpyrazin	2-($^2H_5$)ethyl-3,6-dimethylpyrazine	135	139-141	I	y = 1.214x + -0.0515	0.9979
2-methoxyphenol	2-($^2H_3$)methoxyphenol	124	127	I	y = 1.1161x + -0.0538	0.9999
dimethyl trisulfide	($^2H_6$)dimethyl trisulfide	126	132	I	y = 1.1778x + -0.012	0.9999
linalool	3($^2H_3$)methyl-7-methyl-(4,4-$^2H_2$)octa-1,6-dien-3-ol	136	141	I	y = 1.1497x + 0.1411	0.9942
ethyl phenylacetate	ethyl ($^2H_5$)phenylacetate	104	109	I	y = 1.0111x + 0.0066	1.0000
sotolone	3-hydroxy-4-methyl-5-(^{13}C)methyl(5-^{13}C)furan-2(5H)-one	128	130	I	y = 0.9564x + 0.0843	0.9982
ethyl-2-methylbutanoat	ethyl 2-($^2H_3$)methylbutanoate,	102	105	II	y = 1.0466x + -0.0316	0.9992
ethyl-3-methylbutanoat	ethyl 3-($^2H_3$)methyl(2,2,3,4,4,4-$^2H_6$)butanoate	57	66	II	y = 1.1259x + -0.0216	0.9997
gamma-nonalacton	5-(4,4,5,5-$^2H_2$)pentyloxolan-2-one	138	142	II	y = 0.7889x + -0.0524	0.9997
2-methyl-3-(methyldithio)-furan	2-methyl-3-(($^2H_3$)methyldithio)furan	160	163	II	y = 1.12x + 0.0058	0.9999
4-methylphenol	4-methyl(2,6-$^2H_2$)phenol	107	109	I	y = 0.7428x + 0.2427	0.9982

[a] quantitation with GC-MS (I) or GC-GC-MS (II); [b] y = peak area standard / peak area analyte; x = amount standard (µg) / amount analyte (µg)

Enumeration of Yeasts and Lactic Acid Bacteria during Fermentation: Yeasts and lactic acid bacteria were determined daily from mixed samples of same variation per independent run during fermentation, in the same way as described by Streule et al. [1]. The sample was mixed with dilution solution and a serial dilution was prepared. Then one ml of the dilutions was surface plated on 3M™ Petrifilm® Rapid Yeast and Mold Count Plates for yeasts and on 3M™ Petrifilm® Lactic Acid Bacteria Count Plates for LAB (both from 3M Food Safety, St. Paul, MN, USA). All plates were incubated at room temperature for 2 to 4 days. Results are shown in Tables S2 and S3.

Table S2. Cell counts of yeasts (log cfu/g) during fermentation days (d0, d2, d3, d4, d5, d6, d7) of samples with 1 (PS1) or 3 (PS3) days pod storage, without (D0) or with (D1) depulping and fermented for 3, 4, 5, 6 or 7 days (d) from experimental runs A (*n* = 2), B (*n* = 2; **n* = 1) and C (*n* = 3,**n* = 2).

		Cell Counts Yeasts (log cfu/g)							
Variation		d0	d1	d2	d3	d4	d5	d6	d7 [1]
	PS1D1d3	5.7±0.1	7.8±0.0	7.4±0.2	7.7±0.0	-	-	-	-
Run	PS1D1d4	5.5±0.0	7.8±0.2	7.6±0.2	7.5±0.4	8.3±1.9	-	-	-
A	PS1D1d5	5.6±0.4	7.5±0.1	7.4±0.0	7.3±0.2	7.5±1.2	6.0±1.0	-	-
	PS1D0d5	6.3±0.3	7.6±0.1	7.1±0.2	7.3±0.1	7.3±1.9	5.2±1.4	-	-
	PS1D0d6	5.8±0.1	7.7±0.0	8.4±1	7.9±0.7	6.7±0.1	6.4±1.3	2.8±0.8	6.00*
Run	PS1D1d6	5.5±0.6	8.6±1.2	8.9±1.8	7.9±0.8	8.5±0.7	7.9±0.9	4.6±0.4	6.70*
B	PS3D0d6	6.2±0.7	9.1±1.0	9.1±2.1	7.4±2.3	8.3±0.2	7.4±0.1	4.2±1.7	5.80*
	PS3D1d6	7.0±1.6	8.1±1.3	8.1±1.2	8.8±2.5	6.9±0.8	6.6±0.6	4.9±0	6.60*
	PS1D0d5	5.4±0.7	7.5±0.7	7.2±0.1	6.0±0.3	6.1±0.9	6.2±1.2	-	-
Run	PS1D1d5	5.7±0.3	7.4±0.5	6.9±0.7	6.3±0.6*	6.7±0.2	6.7±0.7	-	-
C	PS1D0d7	5.1±0.6	7.4±0.5	6.8±0.5	5.0±0.7	5.6±1.2	6.2±1.0	6.7±0.6	6.9±0.5
	PS1D1d7	5.0±0.6	7.5±0.6	7.0±0.3	6.1±0.0*	6.8±0.3	6.9±0.4	6.7±0.9	6.9±0.2

[1] Experimental run B: Run 1 started at 7.30 pm at d0 and was fermented until d7 (8.15 am), run 2 started earlier (3 pm), therefore, the last analysis was done at d6 (8 am) and the drying started late afternoon (4 pm), resulting in **n* = 2 at d7.

Table S3. Cell counts of lactic acid bacteria (log cfu/g) during fermentation days (d0, d2, d3, d4, d5, d6, d7) and of samples with 1 (PS1) or 3 (PS3) days pod storage, without (D0) or with (D1) depulping and fermented for 3, 4, 5, 6 or 7 days (d) from experimental runs A (*n* = 4), B (*n* = 4; **n* = 2) and C (*n* = 3).

		Cell Counts Lactic Acid Bacteria (log cfu/g)							
Variation		d0	d1	d2	d3	d4	d5	d6	d7 [1]
	PS1D1d3	5.1±0.1	6.1±0.7	6.9±1.1	7.1±1.7	-	-	-	-
Run	PS1D1d4	4.6±0.4	4.8±0.7	7.0±2.1	7.5±1.3	7.5±0.4	-	-	-
A	PS1D1d5	5.5±0.7	6.3±1.9	6.7±0.8	7.7±1.5	6.9±0.9	6.9±0.5	-	-
	PS1D0d5	4.9±0.1	6.3±0.6	6.2±1.4	7.0±1.6	7.2±0.1	6.4±0.9	-	-
	PS1D0d6	4.4±0.6	5.9±0.6	7.4±0.4	8.8±0.6	7.4±0.2	6.0±0.1	5.3±0.6	7.20*
Run	PS1D1d6	4.9±0.7	6.4±1.3	7.6±0.7	8.0±0.2	8.6±0.4	7.1±0.1	7.1±0.0	7.30*
B	PS3D0d6	5.5±1.8	8.1±1.0	8.1±0.2	8.7±0.7	7.3±1.1	5.7±1.0	6.0±0.2	7.20*
	PS3D1d6	6.1±1.7	7.4±0.2	8.1±0.2	7.6±0.9	6.6±1.3	6.5±0.7	7.4±0.1	6.40*
	PS1D0d5	5.1±1.9	7.4±0.4	8.4±0.4	8.1±0.3	8.0±0.2	8.5±0.4	-	-
Run	PS1D1d5	5.3±0.8	7.7±0.3	8.0±0.3	7.5±0.6	8.2±0.5	8.5±0.8	-	-
C	PS1D0d7	4.6±0.1	7.5±0.6	8.5±0.1	7.6±1.0	8.3±0.3	7.3±2.3	8.5±0.2	8.5±0.4
	PS1D1d7	4.9±1.3	7.6±0.4	7.8±0.7	8.0±0.9	8.3±0.6	8.2±0.5	8.0±0.4	8.0±0.7

[1] Experimental run B: Run 1 started at 7.30 pm at d0 and was fermented until d7 (8.15 am), run 2 started earlier (3 pm), therefore, the last analysis was done at d6 (8 am) and the drying started late afternoon (4 pm), resulting in **n* = 2 at d7.

Reference

[1] Streule, S.; Freimüller Leischtfeld, S.; Galler, M.; Motzer, D.; Poulose-Züst, M.; Miescher Schwenninger, S. Variations in Ecuadorian Cocoa Fermentation and Drying at Two Locations: Implications for Quality and Sensory. *Foods* 2024, 13, 137.
